# Intestinal Protists in Captive Non-human Primates and Their Handlers in Six European Zoological Gardens. Molecular Evidence of Zoonotic Transmission

**DOI:** 10.3389/fvets.2021.819887

**Published:** 2022-01-04

**Authors:** Pamela C. Köster, Eva Martínez-Nevado, Andrea González, María T. Abelló-Poveda, Hugo Fernández-Bellon, Manuel de la Riva-Fraga, Bertille Marquet, Jean-Pascal Guéry, Tobias Knauf-Witzens, Annika Weigold, Alejandro Dashti, Begoña Bailo, Elena Imaña, Aly S. Muadica, David González-Barrio, Francisco Ponce-Gordo, Rafael Calero-Bernal, David Carmena

**Affiliations:** ^1^Parasitology Reference and Research Laboratory, Spanish National Centre for Microbiology, Madrid, Spain; ^2^Madrid Zoo Aquarium, Madrid, Spain; ^3^Santillana Zoological Garden, Santillana del Mar, Spain; ^4^Barcelona Zoological Garden, Barcelona, Spain; ^5^Faunia, Madrid, Spain; ^6^La Vallée des Singes Zoological Park, Le Gureau, France; ^7^Wilhelma Zoological-Botanical Garden, Stuttgart, Germany; ^8^Departamento de Ciências e Tecnologia, Universidade Licungo, Zambézia, Mozambique; ^9^Department of Microbiology and Parasitology, Faculty of Pharmacy, Complutense University of Madrid, Madrid, Spain; ^10^Salud Veterinaria y Zoonosis (SALUVET), Department of Animal Health, Faculty of Veterinary, Complutense University of Madrid, Madrid, Spain

**Keywords:** protists, intestinal parasites, commensals, captive non-human primates, genotyping, conservation, zoonosis, transmission

## Abstract

We assessed the occurrence, genetic diversity, and zoonotic potential of four protozoan (*Cryptosporidium* spp., *Entamoeba histolytica, Entamoeba dispar, Giardia duodenalis*), one stramenopile (*Blastocystis* sp.), one microsporidia (*Enterocytozoon bieneusi*), and two ciliate (*Balantioides coli, Troglodytella abrassarti*) intestinal parasite or commensal protist species in captive non-human primates (NHP) and their zookeepers from six European zoological gardens in France (*n* = 1), Germany (*n* = 1), and Spain (*n* = 4). Faecal samples from NHP (*n* = 454) belonging to 63 species within 35 genera and humans (*n* = 70) were collected at two sampling periods in each participating institution between October 2018-August 2021. Detection and species identification was accomplished by PCR and Sanger sequencing of the *ssu* rRNA and/or ITS genes. Sub-genotyping analyses using specific markers were conducted on isolates positive for *G. duodenalis* (*gdh, bg, tpi*) and *Cryptosporidium* spp. (*gp60*). Overall, 41.0% (186/454) and 30.0% (21/70) of the faecal samples of NHP and human origin tested positive for at least one intestinal protist species, respectively. In NHP, *Blastocystis* sp. was the most prevalent protist species found (20.3%), followed by *G. duodenalis* (18.1%), *E. dispar* (7.9%), *B. coli* and *T. abrassarti* (1.5% each), and *Cryptosporidium* spp. and *E. bieneusi* (0.9% each). Occurrence rates varied largely among NHP host species, sampling periods, and zoological institutions. The predominant protist species found in humans was *Blastocystis* sp. (25.7%), followed by *Cryptosporidium* spp. (2.9%), *E. dispar* (1.4%), and *G. duodenalis* (1.4%). Sequencing of PCR-positive amplicons in human and/or NHP confirmed the presence of *Cryptosporidium* in six isolates (*C. hominis*: 66.7%, *C. parvum*: 33.3%), *G. duodenalis* in 18 isolates (assemblage A: 16.7%, assemblage B: 83.3%), *Blastocystis* in 110 isolates (ST1:38.2%, ST2:11.8%, ST3: 18.2%, ST4: 9.1%, ST5: 17.3%, ST8: 2.7%, ST13: 0.9%), and *E. bieneusi* in four isolates (CM18: 75.0%, Type IV: 25.0%). Zoonotic transmission events involving *Blastocystis* ST1–ST4 were identified in four zoological institutions. Zoonotic transmission of *C. hominis* was highly suspected, but not fully demonstrated, in one of them. Monitoring of intestinal protist species might be useful for assessing health status of captive NHP and their zookeepers, and to identify transmission pathways of faecal-orally transmitted pathogens.

## Introduction

Diarrhoea is one of the leading problems requiring veterinary care in captive non-human primates (NHP). Gastrointestinal manifestations may arise as consequence of nutritional changes, stress and/or infection by viral, bacterial and parasitic pathogens (1). Among the latter, the protozoa *Entamoeba histolytica, Giardia duodenalis* (syn. *G. intestinalis, G. lamblia*), *Cryptosporidium* spp., and *Balantioides coli* have been often associated with symptomatic infections, leading to a wide range of gastrointestinal disorders and even severe disease and death (2–5). Other eukaryotic microorganisms frequently found in captive NHP, such as the stramenopile *Blastocystis* sp. or, to a lesser extent, the microsporidia *Enterocytozoon bieneusi* are recognised as potential pathogens in humans (6, 7), but their veterinary health significance remains to be elucidated. Finally, some protist species, such as *Entamoeba dispar* or the ape-restricted *Troglodytella abrassarti*, are non-pathogenic commensals. However, diagnostic differentiation between pathogenic *E. histolytica* and non-pathogenic members of the *Entamoeba* complex (including, but not limited to, *E. dispar*) is important as these species occur sympatrically and their developmental stages are morphologically indistinguishable (8). The entodiniomorphid ciliate *T. abrassarti* is thought to participate in colonic fermentation of fibre during digestion, so changes in diet may determine its abundance in the intestinal tract of captive NHP (9). In all cases, transmission is primarily through the faecal-oral route via direct contact with infected hosts (or their faecal material), or indirect through the ingestion of contaminated water or food.

Morphological identification by microscopy examination remains the reference method for parasitological diagnosis on faecal samples (10). However, this approach is labour-intensive, requires well-trained microscopists, and lacks diagnostic sensitivity. Highly sensitive molecular methods based on PCR and Sanger sequencing not only improves drastically the diagnostic performance of conventional microscopy (11), but also allows the differentiation of pathogenic species and genotypes circulating in a given host species and a defined epidemiological scenario. In the case of captive NHP, genotyping and sub-genotyping methods are particularly useful to ascertain the molecular diversity of intestinal parasites. This information is essential when assessing the frequency and directionality of zoonotic transmission events between resident animals and their caretakers (12, 13).

Intestinal protist parasites differ in intra-species generic diversity. Thus, *E. histolytica* has a rather low level of single nucleotide diversity (14), although certain genetic variants of the parasite may be involved in virulence and disease outcome (15). In contrast, *G. duodenalis* (the only *Giardia* species infective to NHP and humans) consists of eight (A–H) genotypes known as assemblages based on sequence analysis of several genes, of which assemblages A and B are considered zoonotic (16). To date, no association between *G. duodenalis* genotypes and the occurrence of diarrhoea have been conclusively demonstrated in human infections (17). The genus *Cryptosporidium* comprises no less than 45 valid species (18), with *C. hominis* causing most of the infections reported in NHP and humans globally (19). Using the 60 kDa glycoprotein (*gp60*) as genotyping marker, NHP are susceptible to infections by nine (Ia, Ib, Id, Ie, If, Ii, Ik, Im, and In) *C. hominis* genotype families. Of them, Ia–If have been consistently reported in human populations (19), whereas Ii, Im, and In families seem NHP-adapted and are very rarely or not at all seen in humans (20). A wide genetic diversity has also been identified within *Blastocystis* sp. based on nucleotide polymorphism at the small subunit ribosomal RNA gene (*ssu* rRNA), resulting in the identification of at least 28 subtypes (ST1–ST17, ST21, and ST23-ST32) with marked differences in host range and specificity (21, 22). Of them, zoonotic ST1–ST5, ST7–ST10, and non-zoonotic ST11, ST13, ST15, and ST19 have been identified in captive and wild NHP globally (23). More than 500 genotypes have been defined within *E. bieneusi* based on the ribosomal internal transcribed spacer (ITS) region and grouped in 11 phylogenetic groups, of which Group 1 and Group 2 include most of the potentially zoonotic genotypes of the parasite (24). Common *E. bieneusi* genotypes shared by NHP and humans include genotypes A, BEB4, BEB6, D, EbpA, EbpC, I, J, and Type IV (24). Comparatively, far less information is currently available on the genetic diversity of the ciliates *B. coli* and *T. abrassarti* (25, 26).

This study is part of a large research project aiming at investigating the occurrence, molecular diversity, and zoonotic transmission of potential diarrhoea-causing intestinal protist species in captive, semi-captive, and wild NHP populations under different epidemiological scenarios in Africa, Europe, and South America. Our previous studies focused on wild chimpanzees in Côte d'Ivoire (27) and Senegal (28), and captive/semi-captive NHP in Côte d'Ivoire, Peru, Sierra Leona, and Spain (13, 29). The present study completes the series investigating captive NHP and their caretakers in six European zoological gardens from France, Germany, and Spain.

## Materials and Methods

### Ethical Statement

This study was approved by Ethics Committee of the Health Institute Carlos III under the reference number CEI PI 90_2018-v2. Written informed consent was obtained from zookeepers that volunteered to participate in the survey. This study was carried out in accordance with the International Guiding Principles for Biomedical Research Involving Animals issued by the Council for International Organisation of Medical Sciences and the International Council for Laboratory Animal Science (RD 53/2013).

### Study Design

We conceived this investigation as an observational, transversal, molecular-based epidemiological study with two sampling periods to allow for assessing temporality as driver of variation in parasitism. This study was considered of special interest and prioritised by the Great Ape Taxon Advisory Group of the European Association of Zoos and Aquaria (EAZA). Taking advantage of this, we approached four Spanish (Barcelona Zoo, Faunia, Madrid Zoo Aquarium, Santillana Zoo), one French (La Vallée des Singes), and one German (Wilhelma Zoological-Botanical Garden) zoological institutions and invited them to participate in the survey. Husbandry in all participating zoological institutions was according the EAZA Best Practise Guidelines for each species or similar, providing the best possible care with good levels of welfare and with sanitary safety for animals, staff and visitors. Employees and visiting staff working with NHP wore personal protective equipment when in contact with the animals or their faecal material.

We requested from participating zoos the provision of faecal samples from their resident non-human primates (NHP) in a representative manner, and their handlers (zookeepers, veterinarians, researchers) to allow for assessing the potential occurrence of zoonotic transmission events between humans and NHP.

### Faecal Sample Collection

We collected 454 fresh faecal samples from NHP belonging to 63 species within 35 genera, and 70 fresh faecal samples from humans (Supplementary Table 1). We collected faecal samples from NHP (5–10 g from the inner core) directly from the ground at the time of routine cleaning and sanitation of enclosures. We transferred faecal specimens to sterile polystyrene plastic flasks and recorded information regarding sex, age, faecal consistency, and enclosure sharing with other NHP species when available. Collected faecal samples could not always be linked to individual NHP.

We provided volunteer zoo handlers with sampling kits including uniquely labelled sterile polystyrene plastic flask with spatula, informed consent, and a standardised questionnaire (Supplementary Table 2). Questions included sociodemographic characteristics (e.g., age, sex), behavioural habits (e.g., hand and fruit/vegetable washing, diarrhoea in the participant or close relatives, having pets, travelling abroad), work-related potential risk factors (e.g., contact with faecal material from NHP and/or other animal species, being a food handler), and use of drinking/recreational water.

NHP or human faecal samples were shipped without any preservative at 4°C (≤ 72 h from collection) or −20°C (>72 h from collection) to the Parasitology Reference and Research Laboratory, Spanish National Centre for Microbiology, Majadahonda (Spain) for further processing and downstream molecular testing.

### Sampling at the Barcelona Zoo

We collected faecal samples (*n* = 79) from captive NHP and zoo handlers (*n* = 7) at the Barcelona Zoo (BZ, Barcelona, Spain) in March 2019 and March 2020 (Tables 1, 2). The BZ is a city zoo of 13 ha that keeps 1,921 specimens of 246 species which includes 107 primate individuals belonging to six families (Atelidae, Cebidae, Cercopithecidae, Hominidae, Hylobatidae, and Lemuridae). All the individuals were kept in single-species social groups except for the orangutans who share their facility with two gibbons. Collected faecal samples belonged to eight different NHP genera including *Ateles* (*n* = 5), *Cercocebus* (*n* = 5), *Gorilla* (*n* = 27), *Lemur* (*n* = 5), *Macaca* (*n* = 4), *Mandrillus* (*n* = 6), *Pan* (*n* = 15), and *Pongo* (*n* = 12).

**Table 1:** Frequency of intestinal protist species detected in captive non-human primates by participating institution and sampling period in the present study.

			***Cryptosporidium*** **spp**.		***E. histolytica***		***E. dispar***		***G. duodenalis***		***Blastocystis*** **sp**.		***E. bieneusi***		***B. coli***		***T. abrassarti***	
**Institution**	**Sampling period**	**Samples (*n*)**	**Pos**.	**%**	**Pos**.	**%**	**Pos**.	**%**	**Pos**.	**%**	**Pos**.	**%**	**Pos**.	**%**	**Pos**.	**%**	**Pos**.	**%**
Barcelona	1	34	0	0.0	0	0.0	7	20.6	14	41.2	5	14.7	1	2.9	0	0.0	0	0.0
Zoo	2	45	0	0.0	0	0.0	14	31.1	9	20.0	18	40.0	0	0.0	0	0.0	0	0.0
	Both	79	0	0.0	0	0.0	21	26.6	23	29.1	23	29.1	1	1.3	0	0.0	0	0.0
Faunia	1	38	0	0.0	0	0.0	0	0.0	9	23.7	6	15.8	0	0.0	0	0.0	0	0.0
	2	28	0	0.0	0	0.0	0	0.0	0	0.0	2	7.1	0	0.0	0	0.0	0	0.0
	Both	66	0	0.0	0	0.0	0	0.0	9	13.6	8	12.1	0	0.0	0	0.0	0	0.0
Madrid Zoo	1	62	1	1.6	0	0.0	0	0.0	11	17.7	25	40.3	0	0.0	5	8.1	0	0.0
Aquarium	2	31	0	0.0	0	0.0	0	0.0	5	16.1	13	41.9	1	3.2	2	6.5	0	0.0
	Both	93	1	1.1	0	0.0	0	0.0	16	17.2	38	40.9	1	1.1	7	7.5	0	0.0
Santillana Zoo	1	31	3	9.7	0	0.0	2	6.5	5	16.1	5	16.1	0	0.0	0	0.0	0	0.0
	2	55	0	0.0	0	0.0	8	14.5	9	16.4	14	25.5	0	0.0	0	0.0	0	0.0
	Both	86	3	3.5	0	0.0	10	11.6	14	16.3	19	22.1	0	0.0	0	0.0	0	0.0
La Vallée des	1	44	0	0.0	0	0.0	4	9.1	4	9.1	1	2.3	1	2.3	0	0.0	0	0.0
Singes	2	40	0	0.0	0	0.0	1	2.5	4	10.0	0	0.0	1	2.5	0	0.0	0	0.0
	Both	84	0	0.0	0	0.0	5	6.0	8	9.5	1	1.2	2	2.4	0	0.0	0	0.0
Wilhelma Zoological-Botanical Garden	1	18	0	0.0	0	0.0	0	0.0	3	16.7	0	0.0	0	0.0	0	0.0	4	22.2
	2	28	0	0.0	0	0.0	0	0.0	9	32.1	3	10.7	0	0.0	0	0.0	3	10.7
	Both	46	0	0.0	0	0.0	0	0.0	12	26.1	3	6.5	0	0.0	0	0.0	7	15.2
Total		454	4	0.9	0	0.0	36	7.9	82	18.1	92	20.3	4	0.9	7	1.5	7	1.5

**Table 2:** Frequency of intestinal protist species detected by primate genera in the present study.

		***Cryptosporidium*** **spp**.		***E. histolytica***		***E. dispar***		***G. duodenalis***		***Blastocystis*** **sp**.		***E. bieneusi***		***B. coli***		***T. abrassarti***	
**Genus**	**Samples (*n*)**	**Pos**.	**%**	**Pos**.	**%**	**Pos**.	**%**	**Pos**.	**%**	**Pos**.	**%**	**Pos**.	**%**	**Pos**.	**%**	**Pos**.	**%**
*Alouatta*	4	0	0.0	0	0.0	1	25.0	1	25.0	0	0.0	0	0.0	0	0.0	0	0.0
*Aotus*	6	0	0.0	0	0.0	0	0.0	0	0.0	1	16.7	0	0.0	0	0.0	0	0.0
*Ateles*	10	0	0.0	0	0.0	5	50.0	2	20.0	2	20.0	0	0.0	0	0.0	0	0.0
*Callicebus*	3	0	0.0	0	0.0	1	33.3	0	0.0	1	33.3	0	0.0	0	0.0	0	0.0
*Callimico*	6	0	0.0	0	0.0	0	0.0	2	33.3	0	0.0	0	0.0	0	0.0	0	0.0
*Callithrix*	11	1	9.1	0	0.0	0	0.0	4	36.4	0	0.0	0	0.0	0	0.0	0	0.0
*Cebuella*	7	0	0.0	0	0.0	0	0.0	2	28.6	0	0.0	0	0.0	0	0.0	0	0.0
*Cebus*	27	0	0.0	0	0.0	0	0.0	3	11.1	0	0.0	0	0.0	0	0.0	0	0.0
*Cercocebus*	5	0	0.0	0	0.0	3	60.0	0	0.0	5	100	0	0.0	0	0.0	0	0.0
*Cercopithecus*	7	0	0.0	0	0.0	0	0.0	0	0.0	4	57.1	0	0.0	0	0.0	0	0.0
*Colobus*	13	0	0.0	0	0.0	0	0.0	0	0.0	10	76.9	0	0.0	0	0.0	0	0.0
*Eulemur*	12	0	0.0	0	0.0	0	0.0	3	25.0	0	0.0	0	0.0	0	0.0	0	0.0
*Galago*	2	0	0.0	0	0.0	0	0.0	1	50.0	0	0.0	0	0.0	0	0.0	0	0.0
*Gorilla*	56	0	0.0	0	0.0	1	1.8	15	26.8	5	8.9	2	3.6	1	1.8	2	3.6
*Homo*	70	2	2.9	0	0.0	1	1.4	1	1.4	18	25.7	0	0.0	0	0.0	0	0.0
*Hylobates*	8	0	0.0	0	0.0	0	0.0	1	12.5	2	25.0	0	0.0	2	25.0	0	0.0
*Lagothrix*	2	0	0.0	0	0.0	0	0.0	0	0.0	0	0.0	0	0.0	0	0.0	0	0.0
*Lemur*	42	0	0.0	0	0.0	1	2.4	16	38.1	15	35.7	0	0.0	0	0.0	0	0.0
*Leontopithecus*	14	0	0.0	0	0.0	1	7.1	1	7.1	0	0.0	0	0.0	0	0.0	0	0.0
*Macaca*	13	1	7.7	0	0.0	4	30.8	1	7.7	8	61.5	0	0.0	0	0.0	0	0.0
*Mandrillus*	18	0	0.0	0	0.0	1	5.6	1	5.6	8	44.4	0	0.0	0	0.0	0	0.0
*Mico*	6	0	0.0	0	0.0	0	0.0	1	16.7	0	0.0	0	0.0	0	0.0	0	0.0
*Nomascus*	2	0	0.0	0	0.0	0	0.0	0	0.0	0	0.0	0	0.0	0	0.0	0	0.0
*Nycticebus*	1	0	0.0	0	0.0	0	0.0	0	0.0	0	0.0	0	0.0	0	0.0	0	0.0
*Pan*	59	1	1.7	0	0.0	3	5.1	16	27.1	12	20.3	0	0.0	4	6.8	5	8.5
*Papio*	11	0	0.0	0	0.0	0	0.0	2	18.2	7	63.6	0	0.0	0	0.0	0	0.0
*Perodicticus*	1	0	0.0	0	0.0	0	0.0	0	0.0	1	100	0	0.0	0	0.0	0	0.0
*Pithecia*	10	0	0.0	0	0.0	2	20.0	1	10.0	0	0.0	0	0.0	0	0.0	0	0.0
*Plecturocebus*	3	0	0.0	0	0.0	0	0.0	0	0.0	0	0.0	0	0.0	0	0.0	0	0.0
*Pongo*	37	0	0.0	0	0.0	12	32.4	4	10.8	10	27.0	0	0.0	0	0.0	0	0.0
*Saguinus*	19	1	5.3	0	0.0	1	5.3	3	15.8	0	0.0	1	5.3	0	0.0	0	0.0
*Saimiri*	14	0	0.0	0	0.0	0	0.0	1	7.1	0	0.0	1	7.1	0	0.0	0	0.0
*Sapajus*	5	0	0.0	0	0.0	0	0.0	0	0.0	0	0.0	0	0.0	0	0.0	0	0.0
*Theropithecus*	3	0	0.0	0	0.0	0	0.0	0	0.0	0	0.0	0	0.0	0	0.0	0	0.0
*Trachypithecus*	1	0	0.0	0	0.0	0	0.0	0	0.0	1	100	0	0.0	0	0.0	0	0.0
*Unknown*	2	0	0.0	0	0.0	0	0.0	0	0.0	0	0.0	0	0.0	0	0.0	0	0.0
*Varecia*	14	0	0.0	0	0.0	0	0.0	1	7.1	0	0.0	0	0.0	0	0.0	0	0.0
Total	524	6	1.1	0	0.0	37	7.1	83	15.8	110	21.0	4	0.8	7	1.3	7	1.3

### Sampling at Faunia

We collected faecal samples (*n* = 66) from captive NHP and zoo handlers (*n* = 15) at Faunia (Madrid, Spain) in November 2018 and November-December 2020 (Tables 1, 2). Faunia is an immersive city zoo of 14 ha in which near 3,000 specimens of more than 300 different species living in habitats designed to mimic natural ecosystems. These include 124 non-ape primates belonging to seven families (Aotidae, Callitrichidae, Cebidae, Galagidae, Lemuridae, Lorisidae, and Pitheciidae). Most NHPs share indoor enclosures with other animal species. Members of the Lemuridae family were kept together in the same enclosure. Collected faecal samples belonged to 13 different NHP genera including *Aotus* (*n* = 6), *Callimico* (*n* = 2), *Callithrix* (*n* = 3), *Cebus* (*n* = 18), *Eulemur* (*n* = 2), *Galago* (*n* = 2), *Lemur* (*n* = 22), *Leontopithecus* (*n* = 1), *Nycticebus* (*n* = 1), *Perodicticus* (*n* = 1), *Pithecia* (*n* = 2), *Saguinus* (*n* = 2), and *Saimiri* (*n* = 4).

### Sampling at the Madrid Zoo Aquarium

We collected faecal samples (*n* = 93) from captive NHP and zoo handlers (*n* = 31) at the Madrid Zoo Aquarium (MZA, Madrid, Spain) in November 2018 and September-October 2019 (Tables 1, 2). The MZA is a city zoo of 21 ha that keeps 1,520 specimens of 336 species, which includes 187 primate individuals belonging to five families (Cebidae, Cercopithecidae, Hominidae, Hylobatidae, and Lemuridae). All the individuals were kept on social groups of their own species, except for the orangutans who share their facility with three gibbons and another facility with members of different genera of the family Lemuridae. Collected faecal samples belonged to 10 different NHP genera including *Cebus* (*n* = 6), *Colobus* (*n* = 7), *Gorilla* (*n* = 14), *Hylobates* (*n* = 5), *Lemur* (*n* = 7), *Mandrillus* (*n* = 8), *Pan* (*n* = 15), *Papio* (*n* = 11), *Pongo* (*n* = 12), and *Varecia* (*n* = 8).

### Sampling at the Santillana Zoo

We collected faecal samples (*n* = 86) from captive NHP and zoo handlers (*n* = 9) at the Santillana Zoo (SZ, Cantabria, Spain) in October 2018 and February 2020 (Tables 1, 2). SZ extends over 7 ha and hosts near 3,600 specimens of 380 species, of which 22 correspond to NHP belonging to six families (Callitrichidae, Cebidae, Cercopithecidae, Hominidae, Lemuridae, and Pitheciidae). All the individuals were kept on social groups of their own species, except for some members of the genera *Callithrix, Cebuella, Leontopithecus, Pithecia, Saimiri*, and *Saguinus* that share enclosures. Collected faecal samples belonged to 17 different NHP genera including *Callimico* (*n* = 3), *Callithrix* (*n* = 1), *Cebuella* (*n* = 4), *Cercopithecus* (*n* = 5), *Colobus* (*n* = 4), *Eulemur* (*n* = 3), *Lemur* (*n* = 6), *Leontopithecus* (*n* = 8), *Macaca* (*n* = 6), *Mandrillus* (*n* = 2), *Mico* (*n* = 4), *Pan* (*n* = 4), *Pithecia* (*n* = 3), *Pongo* (*n* = 10), *Saguinus* (*n* = 13), *Saimiri* (*n* = 4), unknown (*n* = 2), and *Varecia* (*n* = 4).

### Sampling at La Vallée des Singes

We collected faecal samples (*n* = 84) from captive NHP and zoo handlers (*n* = 8) at the La Vallée de Singes (LVS, Romagne, France) between August 2019 and August 2021 (Tables 1, 2). LVS is an immersive zoo of 24 ha in which near 350 primates of 34 different species belonging to eight families (Atelidae, Callitrichidae, Cebidae, Cercopithecidae, Hominidae, Hylobatidae, Lemuridae, and Pitheciidae) are living on naturally wooded islands separated by water moats. Collected faecal samples belonged to 27 different NHP genera including *Alouatta* (*n* = 2), *Ateles* (*n* = 4), *Callicebus* (*n* = 3), *Callithrix* (*n* = 6), *Cebuella* (*n* = 3), *Cebus* (*n* = 3), *Cercopithecus* (*n* = 2), *Colobus* (*n* = 2), *Eulemur* (*n* = 7), *Gorilla* (*n* = 2), *Hylobates* (*n* = 2), *Lagothrix* (*n* = 2), *Lemur* (*n* = 2), *Leontopithecus* (*n* = 4), *Macaca* (*n* = 2), *Mandrillus* (*n* = 2), *Mico* (*n* = 2), *Nomascus* (*n* = 2), *Pan* (*n* = 6), *Pithecia* (*n* = 4), *Plecturocebus* (*n* = 3), *Pongo* (*n* = 1), *Saguinus* (*n* = 4), *Saimiri* (*n* = 5), *Sapajus* (*n* = 5), *Theropithecus* (*n* = 2), and *Varecia* (*n* = 2).

### Sampling at Wilhelma Zoological-Botanical Garden

We collected faecal samples (*n* = 46) from captive NHP at the Wilhelma Zoological-Botanical Garden (WZBG, Stuttgart, Germany) in April 2019 and March-May 2021 (Table 1). No faecal samples of human origin were available from this institution. The WZBG extends over 30 ha and houses 10,100 specimens of near 1,200 animal (including 86 mammalian) species. These include 12 non-ape (families Callimiconidae, Callitrichidae, Cebidae, Cercopithecidae, Colobidae, and Lorisidae) and three ape (families Hominidae and Hylobatidae) primate genera. The apes were not in contact with any other NHP, whereas in some of the other enclosures more than one NHP species were held together with other NHP and/or other animal species. All NHP had access to natural floor including grass and/or bark or wood chips. Collected faecal samples belonged to 14 different NHP genera including *Alouatta* (*n* = 2), *Ateles* (*n* = 1), *Callimico* (*n* = 1), *Callithrix* (*n* = 1), *Gorilla* (*n* = 13), *Hylobates* (*n* = 1), *Leontopithecus* (*n* = 1), *Macaca* (*n* = 1), *Pan* (*n* = 19), *Pithecia* (*n* = 1), *Pongo* (*n* = 2), *Saimiri* (*n* = 1), *Theropithecus* (*n* = 1), and *Trachypithecus* (*n* = 1).

### DNA Extraction and Purification

We isolated genomic DNA from about 200 mg of each faecal specimen by using the QIAamp DNA Stool Mini Kit (Qiagen, Hilden, Germany) according to the manufacturer's instructions, except that samples mixed with InhibitEX buffer were incubated for 10 min at 95°C. Extracted and purified DNA samples were eluted in 200 μL of PCR-grade water and kept at 4°C until further molecular analysis.

### Molecular Detection of *Cryptosporidium* spp.

We assessed the presence of *Cryptosporidiu*m spp. using a nested-PCR protocol to amplify a 587 bp fragment of the *ssu* rRNA gene of the parasite (30). Amplification reactions (50 μL) included 3 μL of DNA sample and 0.3 μM of the primer pairs CR-P1/CR-P2 in the primary reaction and CR-P3/CPB-DIAGR in the secondary reaction (Supplementary Table 3). Both PCR reactions were carried out as follows: one step of 94°C for 3 min, followed by 35 cycles of 94°C for 40 s, 50°C for 40 s, and 72°C for 1 min, concluding with a final extension of 72°C for 10 min.

### Molecular Differential Detection of *Entamoeba histolytica* and *Entamoeba dispar*

We carried out detection and differential diagnosis between pathogenic *E. histolytica* and non-pathogenic *E. dispar* by a real-time PCR (qPCR) method targeting a 172 bp fragment of the gene codifying the *ssu* rRNA gene of the *E. histolytica*/*E. dispar* complex (31, 32). Amplification reactions (25 μL) consisted of 3 μL template DNA, 12.5 pmol of the primer set Ehd-239F/Ehd-88R, 5 pmol of each TaqMan® probe (Supplementary Table 3), and TaqMan® Gene Expression Master Mix (Applied Biosystems, CA, USA). Detection of parasitic DNA was performed on a Corbett Rotor GeneTM 6000 real-time PCR system (Qiagen) using an amplification protocol consisting of an initial hold step of 2 min at 55°C and 15 min at 95°C followed by 45 cycles of 15 s at 95°C and 1 min at 60°C. We included water (no-template) and genomic DNA (positive) controls in each PCR run.

### Molecular Detection and Characterisation of *Giardia duodenalis*

We conducted *G. duodenalis* DNA detection using a qPCR method targeting a 62-bp region of the gene codifying the *ssu* rRNA gene of the parasite (33). Amplification reactions (25 μL) consisted of 3 μL of template DNA, 0.5 μM of each primer Gd-80F and Gd-127R, 0.4 μM of probe (Supplementary Table 3), and 12.5 μL TaqMan® Gene Expression Master Mix (Applied Biosystems). Cycling conditions and data analysis were as described above for the detection of *E. histolytica*/*E. dispar*.

We subsequently assessed *G. duodenalis* isolates that tested positive by qPCR by sequence-based multi-locus genotyping of the genes encoding for the glutamate dehydrogenase (*gdh*) (34), β-giardin (*bg*) (35), and triose phosphate isomerase (*tpi*) (36) proteins of the parasite. We conducted amplifications by semi-nested and nested PCR protocols using specific primer pairs (Supplementary Table 3).

### Molecular Detection and Characterisation of *Blastocystis* sp.

We identified *Blastocysti*s sp. by a direct PCR protocol targeting the *ssu* rRNA gene of the parasite (37). The assay uses the pan-*Blastocystis*, barcode primer pair RD5/BhRDr to amplify a PCR product of ~600 bp. Amplification reactions (25 μL) included 5 μL of template DNA and 0.5 μM of each primer (Supplementary Table 3). Amplification conditions consisted of one-step of 95°C for 3 min, followed by 30 cycles of 1 min each at 94, 59 and 72°C, with an additional 2 min final extension at 72°C.

### Molecular Detection and Characterisation of *Enterocytozoon bieneusi*

We conducted *E. bieneusi* detection by a nested PCR protocol to amplify the internal transcribed spacer (ITS) region as well as portions of the flanking large and small subunit of the ribosomal RNA gene as previously described (38). We used the outer EBITS3/EBITS4 and inner EBITS1/EBITS2.4 primer sets (Supplementary Table 3) to generate a final PCR product of 390 bp, respectively. PCR reactions (50 μL) consisted of 1 μL of template DNA and 0.2 μM of each primer. Cycling conditions for the primary PCR consisted of one step of 94°C for 3 min, followed by 35 cycles of amplification (denaturation at 94°C for 30 s, annealing at 57°C for 30 s, and elongation at 72°C for 40 s), with a final extension at 72°C for 10 min. Conditions for the secondary PCR were identical to the primary PCR except only 30 cycles were carried out with an annealing temperature of 55°C.

### Molecular Detection of *Balantioides coli*

We attempted *B. coli* detection by a direct PCR assay to amplify the complete ITS1–5.8s-rRNA–ITS2 region and the last 117 bp (3' end) of the *ssu*-rRNA sequence of this ciliate using the primer set B5D/B5RC (39). PCR reactions (25 μL) consisted of 2 μL of template DNA and 0.4 μM of each primer (Supplementary Table 3). PCR conditions were as follows: 94°C for 10 min; 30 cycles of 94°C for 1 min, 60°C for 1 min, 72°C for 1 min, and a final extension for 5 min at 72°C.

### Molecular Detection of *Troglodytella* spp.

Hitherto, we aimed to detect the ciliate mutualist *Troglodytella* spp. by a direct PCR method targeting a 401 bp fragment of the ITS region of the rDNA (ITS1-5.8S rDNA-ITS2) (26). PCR reactions (25 μL) contained 2 μL of template DNA and 0.8 μM of each primer ssu-end/LSU-start (Supplementary Table 3). Conditions of PCR for ITS amplification were initial denaturation for 2 min at 94°C, 35 cycles of 45 s at 94°C, 45 s at 50°C, and 90 s at 72°C, and terminal elongation for 5 min at 72°C.

### PCR and Gel Electrophoresis Standard Procedures

We carried out all the direct, semi-nested, and nested PCR protocols described above on a 2720 Thermal Cycler (Applied Biosystems). Reaction mixes included 2.5 units of MyTAQTM DNA polymerase (Bioline GmbH, Luckenwalde, Germany), and 5 × MyTAQTM Reaction Buffer containing 5 mM dNTPs and 15 mM MgCl_2_. The specific DNA primer and probe sequences used in the present study were detailed in Supplementary Table 3. We routinely used laboratory-confirmed positive and negative DNA samples of human and animal origin for each parasitic species investigated as controls and included them in each round of PCR. We visualised PCR amplicons on 1.5–2% D5 agarose gels (Conda, Madrid, Spain) stained with Pronasafe (Conda) nucleic acid staining solutions. We used a 100 bp DNA ladder (Boehringer Mannheim GmbH, Baden-Wurttemberg, Germany) for the sizing of obtained amplicons.

### Sequence Analyses

We directly sequenced positive-PCR products in both directions using appropriate internal primer sets (Supplementary Table 3). We conducted DNA sequencing by capillary electrophoresis using the BigDye® Terminator chemistry (Applied Biosystems) on an ABI PRISM 3130 automated DNA sequencer. We visually inspected the obtained chromatograms for quality control and for detecting the presence of ambiguous (double peak) positions. Sequences obtained in this study were deposited in GenBank under accession numbers OK285278–OK285280 (*Cryptosporidium* spp.), OK318919–OK318938 and OL456212 (*Giardia duodenalis*), OK285223–OK285250 (*Blastocystis* sp.), OK533569– OK533571 and OL458611 (*Enterocytozoon bieneusi*), OK493778 (*Balantioides coli*), and OK493782 (*Troglodytella abrassarti*).

## Results

We present in Supplementary Table 1 the full dataset including diagnostic and molecular genotyping results in NHP and humans from the six European zoological institutions that participated in the present study.

### Occurrence of Intestinal Protist Species in NHP

Overall, 41.0% (186/454) of the NHP faecal samples that we examined tested positive for at least one intestinal protist species. We identified *Blastocystis* sp. as the most prevalent protist species found (20.3%, range: 1.2–40.9%), followed by *G. duodenalis* (18.1%, range: 9.5–29.1%), *E. dispar* (7.9%, range: 0.0–26.6%), *B. coli* (1.5%, 0.0–7.5%), *T. abrassarti* (1.5%, 0.0–15.2%), *Cryptosporidium* spp. (0.9%, 0.0–3.5%), and *E. bieneusi* (0.9%, 0.0–2.4%). *Entamoeba histolytica* was not detected in any of the zoological institutions participating in the study (Table 1).

**Figure 1:**
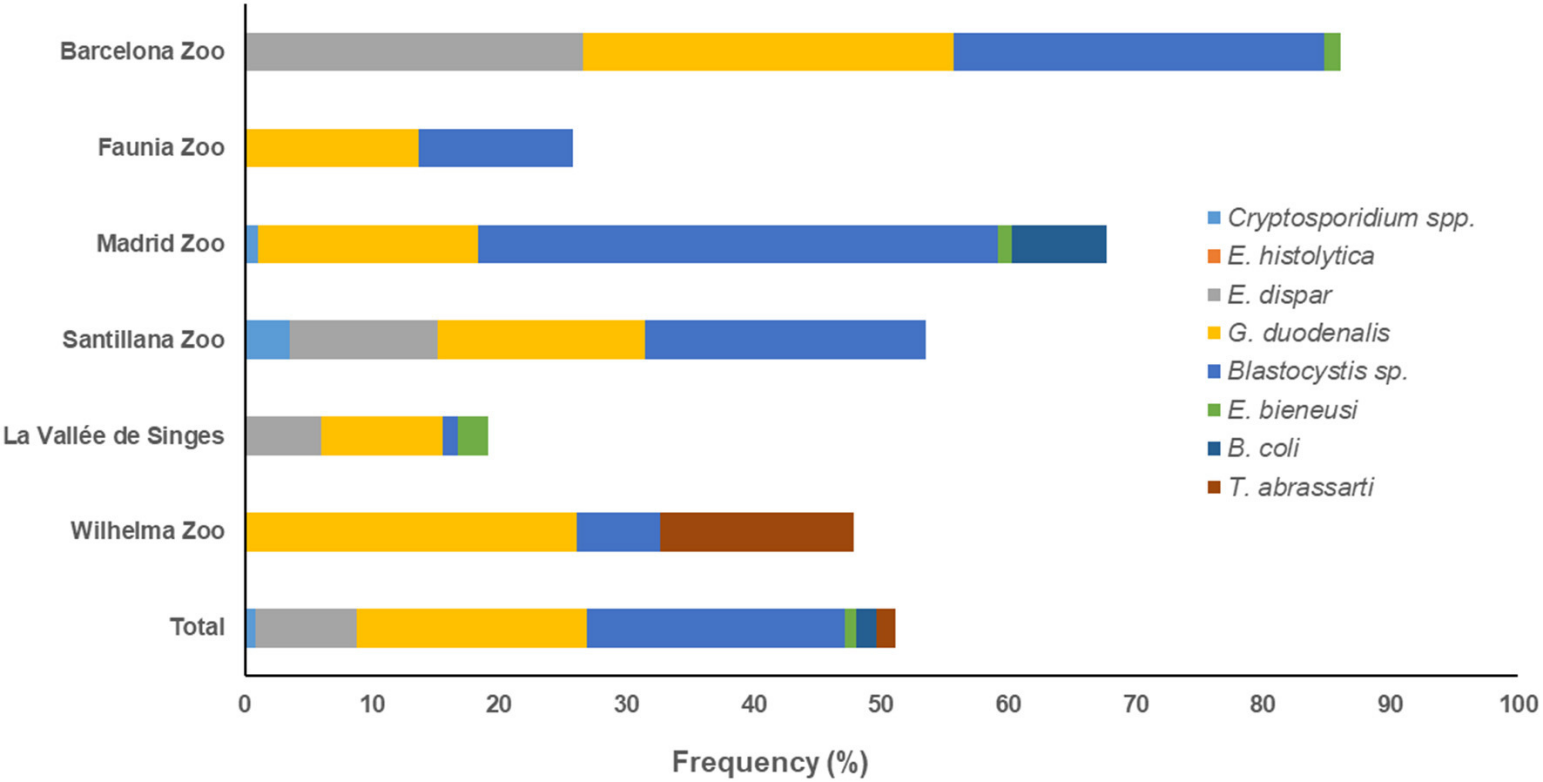
Accumulated frequencies of enteric protist species detected in captive non-human primates by participating institution in the present study.

According to the zoological institution of origin, both *G. duodenalis* and *Blastocysti*s sp. were the protist species most commonly found in captive NHP in BZ (29.1% each) and Faunia (13.6 and 12.1%, respectively). *Blastocystis* sp. was the most prevalent protist species in MZA (40.9%) and SZ (22.1%), and *G. duodenalis* (26.1%) in WZBG. Finally, both *G. duodenalis* and *E. dispar* were the most commonly found protist species in LVS (9.5 and 6.0%, respectively) (Figure 1).

We observed marked differences on the distribution of protist species among the participating zoological institutions. Whereas *G. duodenalis* and *Blastocystis* sp. were present in all six institutions investigated, we detected *Cryptosporidium* spp. only in MZA and SZ, *E. bieneusi* only in BZ, MZA, and LVS, *B. coli* only in MZA, and *T. abrassarti* only in WZBG (Figure 1).

According to host, we found *Cryptosporidium* spp. in four NHP genera (equally present in *Callithrix, Macaca, Pan*, and *Saguinus*), *E. dispar* in 13 NHP genera (with *Ateles, Macaca*, and *Pongo* accounting for 58.3% of the isolates), *G. duodenalis* in 22 NHP genera (with *Gorilla, Lemur*, and *Pan* accounting for 57.3% of the isolates), and *Blastocystis* sp. in 16 NHP genera (with *Colobus, Lemur, Pan*, and *Pongo* accounting for 51.1% of the isolates). We detected *E. bieneusi* only in NHP of the genera *Gorilla, Saguinus*, and *Saimiri*, and *B. coli* in NHP of the genera *Gorilla, Hylobates*, and *Pan*. Finally, we observed *T. abrassarti* only in members of the genera *Gorilla* and *Pan* (Table 2).

Most of the protist species found in NHP faecal samples were observed as mono-infections/colonisations (76.9%, 143/186), with *G. duodenalis* (31.2%, 58/186) and *Blastocystis* sp. (30.1%, 56/186) accounting for the bulk of them. Co-infections/colonisations involving two protist species were observed in 21.5% (40/186) of the faecal samples analysed, being *Blastocystis* sp. + *G. duodenalis* (8.1%, 15/186) and *Blastocystis* sp. + *E. dispar* (6.9%, 13/186) the most frequent combinations found. A triple infection/colonisation involving *Blastocystis* sp. + *E. dispar* + *G. duodenalis* was found in three faecal samples (1.6%, 3/186) (Supplementary Table 4).

### Occurrence of Intestinal Protist Species in Humans

Overall, 30.0% (21/70) of the human faecal samples that we examined tested positive for at least one intestinal protist species. Observed infection/colonisation rates were of 28.6% (2/7) at BZ, 26.7% (4/15) at Faunia, 35.5% (11/31) at MZA, 12.5% (1/8) at LVS, and 33.3% (3/9) at SZ. No faecal samples from human origin were available from WZBG (Table 3).

**Table 3:** Frequency of intestinal protist species detected in zookeepers handling non-human primates by participating institution and sampling period in the present study.

			***Cryptosporidium*** **spp**.		***E. histolytica***		***E. dispar***		***G. duodenalis***		***Blastocystis*** **sp**.		***E. bieneusi***		***B. coli***	
**Institution**	**Sampling period**	**Samples (*n*)**	**Pos**.	**%**	**Pos**.	**%**	**Pos**.	**%**	**Pos**.	**%**	**Pos**.	**%**	**Pos**.	**%**	**Pos**.	**%**
Barcelona Zoo	1	5	0	0.0	0	0.0	0	0.0	0	0.0	2	40.0	0	0.0	0	0.0
	2	2	0	0.0	0	0.0	0	0.0	0	0.0	0	0.0	0	0.0	0	0.0
	Both	7	0	0.0	0	0.0	0	0.0	0	0.0	2	28.6	0	0.0	0	0.0
Faunia	1	6	0	0.0	0	0.0	0	0.0	0	0.0	2	33.3	0	0.0	0	0.0
	2	9	0	0.0	0	0.0	0	0.0	0	0.0	2	22.2	0	0.0	0	0.0
	Both	15	0	0.0	0	0.0	0	0.0	0	0.0	4	26.7	0	0.0	0	0.0
Madrid Zoo	1	17	2	11.8	0	0.0	0	0.0	1	5.9	8	47.1	0	0.0	0	0.0
Aquarium	2	14	0	0.0	0	0.0	0	0.0	0	0.0	1	7.1	0	0.0	0	0.0
	Both	31	2	6.5	0	0.0	0	0.0	1	3.2	9	29.0	0	0.0	0	0.0
Santillana Zoo	1	3	0	0.0	0	0.0	0	0.0	0	0.0	0	0.0	0	0.0	0	0.0
	2	6	0	0.0	0	0.0	0	0.0	0	0.0	3	50.0	0	0.0	0	0.0
	Both	9	0	0.0	0	0.0	0	0.0	0	0.0	3	33.3	0	0.0	0	0.0
La Vallée des	1	6	0	0.0	0	0.0	1	16.7	0	0.0	0	0.0	0	0.0	0	0.0
Singes	2	2	0	0.0	0	0.0	0	0.0	0	0.0	0	0.0	0	0.0	0	0.0
	Both	8	0	0.0	0	0.0	1	12.5	0	0.0	0	0.0	0	0.0	0	0.0
Total		70	2	2.9	0	0.0	1	1.4	1	1.4	18	25.7	0	0.0	0	0.0

*Wilhelma Zoological-Botanical Garden did not provide stool samples of human origin for this survey. Troglodytella abrassarti was not investigated in human beings because of its ape-specific nature*.

We identified *Blastocystis* sp. as the most prevalent protist species found in human faecal samples (25.7%, range: 0.0–33.3%), followed by *Cryptosporidium* spp. (2.9%, range: 0.0–6.5), *E. dispar* (1.4%, range: 0.0–12.5%), and *G. duodenalis* (1.4%, 0.0–3.2%). *Entamoeba histolytica, E. bieneusi*, and *B. coli* were not detected in any of the human faecal sample analysed (Table 3). *Troglodytella abrassarti* was not investigated in human beings because of its ape-specific nature.

**Figure 2:**
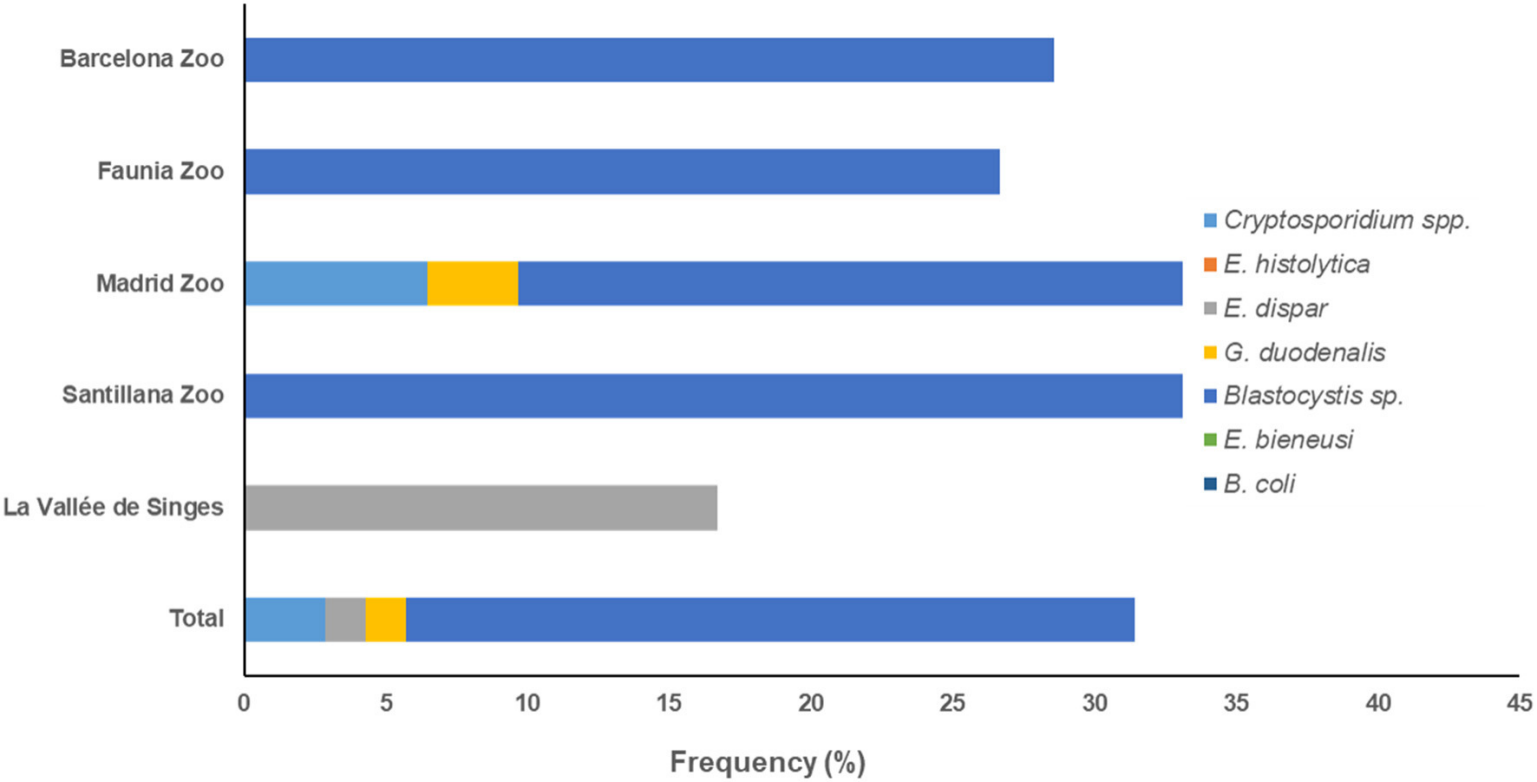
Accumulated frequencies of enteric protist species detected in humans by participating institution in the present study.

According to the zoological institution of origin, *Blastocystis* sp. was the only protist species found circulating in humans in BZ, Faunia, and SZ, and *E. dispar* in LVS. We detected three protist species (*Blastocystis*: 29.0%, *Cryptosporidium* spp.: 6.5%, and *G. duodenalis*: 3.2%) in faecal samples of human origin from the MZA (Figure 2).

The vast majority of the protist species found in human faecal samples were observed as mono-infections/colonisations (95.2%, 20/21), with *Blastocystis* sp. (80.9%, 17/21) as the most frequent agent identified. A double infection involving *Blastocystis* sp. + *Cryptosporidium* spp. was found in a single human faecal sample (4.8%, 1/21) (Supplementary Table 5).

### Sampling Variation of Intestinal Protist Species in NHP and Humans

We observed marked differences in the temporal occurrence of intestinal protist species in NHP among the six zoological institutions participating in this study. At the BZ, both *E. dispar* (20.6 vs. 31.1%) and *Blastocystis* sp. (14.7 vs. 40.0%) were more frequently found during the second sampling campaign than during the first sampling campaign, whereas the opposite trend was observed for *G. duodenalis* (41.2 vs. 20.0%) (Table 1). At Faunia, *G. duodenalis* (23.7%) was only detected during the first sapling campaign, whereas *Blastocystis* carriage was more common in the first than in the second sampling campaign (15.8 vs. 7.1%) (Table 1). At MZA, intestinal protist infection/colonisation rates remained practically constant irrespectively of the sampling period considered. The same trend was observed at the SZ with the exception of *Cryptosporidium* spp., which was detected only in the first sampling campaign (9.7%) (Table 1). Higher occurrence rates of *G. duodenalis* (16.7 vs. 32.1%) and *Blastocystis* sp. (0.0 vs. 10.7%) were observed in the first sampling campaign compared to the second sampling period at the WZBG (Table 1).

Sampling variation of intestinal protists in humans were less evident due to the lower number of stool samples available for analyses (Table 2). We observed a marked reduction in protist occurrence in handlers working at the MZA, where *Cryptosporidium* spp. and *G. duodenalis* were not detected in the second sampling campaign and *Blastocystis* carriage was reduced from 47.1 to 7.1% between the first and the second sampling campaigns (Table 2).

### Molecular Characterisation of Intestinal Protist Species in NHP and Humans

We summarised in Table 4 the distribution of intestinal protist species and genotypes (confirmed by Sanger sequencing) by zoological institution and primate genera obtained in the present study. The in-depth molecular features of the obtained protist isolates including sub-genotyping data, presence of single nucleotide polymorphisms, and GenBank accession numbers of representative sequences are shown in Supplementary Tables 6, 7 (NHP) and Supplementary Table 8 (humans).

**Table 4:** Distribution of protist species and genotypes by institution and primate genera in the present study.

**Protist species**	**Institution**	**Genotype**	**Isolates (*n*)**	**Frequency (%)**	**Primate species (number of isolates)**
*Cryptosporidium* spp.	Madrid Zoo Aquarium	*C. hominis*	3	100	***Homo*** (2), *Pan* (1)
	Santillana Zoo	*C. hominis*	1	33.3	*Callithrix* (1)
		*C. parvum*	2	66.7	*Macaca* (1), *Saguinus* (1)
*Giardia duodenalis*	Barcelona Zoo	AII	2	40.0	*Pan* (2)
		BIV	2	40.0	*Lemur* (2)
		B	1	20.0	*Lemur* (1)
	Faunia	BIV	5	100	*Callithrix* (1), *Lemur* (4)
	Madrid Zoo Aquarium	AI	1	25.0	*Cebus* (1)
		BIV	2	50.0	*Lemur* (2)
		B	1	25.0	*Lemur* (1)
	Santillana Zoo	BIV	2	66.7	*Callimico* (1), *Lemur* (1)
		B	1	33.3	*Lemur* (1)
	La Vallée des Singes	BIV	1	100	*Cebuella* (1)
*Blastocystis* sp.	Barcelona Zoo	ST1	9	36.0	*Cercocebus* (5), *Macaca* (1), *Mandrillus* (2), *Pan* (1)
		ST2	1	4.0	*Macaca* (1)
		ST3	6	24.0	*Ateles* (1), *Gorilla* (1), ***Homo*** (2), *Macaca* (1), *Mandrillus* (1)
		ST4	1	4.0	*Ateles* (1)
		ST5	6	24.0	*Gorilla* (2), *Lemur* (1), *Pongo* (3)
		Unknown	2	8.0	*Lemur* (1), *Macaca* (1)
	Faunia	ST1	1	8.3	***Homo*** (1)
		ST2	1	8.3	***Homo*** (1)
		ST3	1	8.3	*Lemur* (1)
		ST4	6	50.0	*Lemur* (4), ***Homo*** (2)
		ST5	1	8.3	*Lemur* (1)
		ST8	2	16.7	*Aotus* (1), *Perodicticus* (1)
	Madrid Zoo Aquarium	ST1	12	25.5	*Colobus* (2), ***Homo*** (5), *Mandrillus* (4), *Papio* (1)
		ST2	11	23.4	***Homo*** (1), *Pan* (9), *Papio* (1)
		ST3	9	19.1	*Colobus* (3), *Papio* (5), *Pongo* (1)
		ST4	3	6.4	***Homo*** (3)
		ST5	12	25.5	*Colobus* (1), *Gorilla* (2), *Hylobates* (1), *Lemur* (4), *Pongo* (4)
	Santillana Zoo	ST1	20	90.9	*Cercopithecus* (4), *Colobus* (4), ***Homo*** (2), *Lemur* (3), *Macaca* (4), *Pan* (1), *Pongo* (2)
		ST3	2	9.1	***Homo*** (1), *Mandrillus* (1)
	La Vallée des Singes	ST8	1	100	*Callicebus* (1)
	Wilhelma Zoological-Botanical Garden	ST3	2	66.7	*Hylobates* (1), *Pan* (1)
		ST13	1	33.3	*Trachypithecus* (1)
*Enterocytozoon bieneusi*	Barcelona Zoo	Type IV	1	100	*Gorilla* (1)
	Madrid Zoo Aquarium	CM18	1	100	*Gorilla* (1)
	La Vallée des Singes	CM18	2	100	*Saguinus* (1), *Saimiri* (1)
*Balantioides coli*	Madrid Zoo Aquarium	–	7	–	*Gorilla* (1), *Hylobates* (2), *Pan* (4)
*Troglodytella abrassarti*	Wilhelma Zoological-Botanical Garden	–	7	–	*Gorilla* (2), *Pan* (5)

*Members of the genus Homo are shown in bold to identify scenarios where zoonotic transmission events may occur*.

We identified two *Cryptosporidium* species including *C. hominis* (66.7%, 4/6; present in members of the genera *Callithrix, Homo*, and *Pan*), and *C. parvum* (33.3%, 2/6; present in NHP of the genera *Macaca* and *Saguinus*). All *Cryptosporidium*-positive samples were obtained in the MZA and the SZ in Spain (Table 4).

The 83 DNA isolates that tested positive for *G. duodenalis* by qPCR generated cycle threshold (Ct) values ranging from 23.4–41.1 (median: 34.0). Of them, 69.9% (58/83) had Ct values ≥30. Only 21.7% (18/83) of the *G. duodenalis*-positive isolates could be genotyped at any of the three loci (*gdh, bg*, or *tpi*) tested. Of them, 88.9% (16/18) had Ct values <30. Sequence analyses revealed the presence of assemblages A (16.7%, 3/18) and B (83.3%, 15/18). Within assemblage A, we detected sub-assemblage AI in a member of the genus *Cebus* (MZA) and AII in two members of the genus *Pan* (BZ). Most of the assemblage B isolates were obtained from lemurids (80.0%, 12/15), with the remaining three coming from members of the genera *Callimico, Callithrix*, and *Cebuella* (6.7%, 1/15 each). Assemblage B sequences were identified in resident NHP from all zoological institutions except WZBG. Sub-genotyping analysis confirmed the presence of sub-assemblage BIV in 80.0% (12/15) of the assemblage B isolates (Table 4).

We identified six *Blastocystis* subtypes circulating among the surveyed NHP and human populations, including ST1 (38.2%, 42/110), ST2 (11.8%, 13/110), ST3 (18.2%, 20/110), ST4 (9.1%, 10/110), ST5 (17.3%, 19/110), ST8 (2.7%, 3/110), and ST13 (0.9%, 1/110). Two additional isolates (1.8%, 2/110) could not be identified at the ST level. ST1-ST3 and ST5 showed a loose host specificity, being able to infect/colonise a wide range (5–11) of primate (including human) genera. In contrast, most ST4 isolates were found in humans or lemurids, ST8 was identified in less-represented genera including *Aotus, Callicebus*, and *Perodicticus*, and ST13 was only detected in a member of the genus *Trachypithecus*. According to the zoological institution of origin, ST1-ST5 were present in BZ, Faunia, and MZA. ST1 and ST3 were the only *Blastocystis* STs identified at the SZ, whereas ST8 was observed only in Faunia and LVS, and ST13 only in WZBG (Table 4).

We identified two *E. bieneusi* genotypes (Type IV and CM18 in members of the genus *Gorilla*, and CM18 in members of the genera *Saguinus* and *Saimiri*) in the BZ, the MZA, and LVS (Table 4).

Finally, *B. coli* was identified in members of the genera *Gorilla, Hylobates*, and *Pan* resident at the MZA and WZBG, whereas *T. abrassarti* was only detected in NHP of the genera *Gorilla* and *Pan* at the WZBG (Table 4).

### Molecular Evidence of Zoonotic Transmission

We considered evidence of zoonotic transmission the finding of the same protist species and genetic variant circulating simultaneously among NHP and their handlers in the same zoological institution and during the same sampling period. Under these premises, we detected *C. hominis* in two zookeepers and a captive chimpanzee (*Pan troglodytes*) investigated during the first sampling campaign at the MZA (Table 4). Lack of genotyping data at the *gp60* locus precluded us to determine the *gp60* family sub-genotype involved in these three infections.

**Figure 3:**
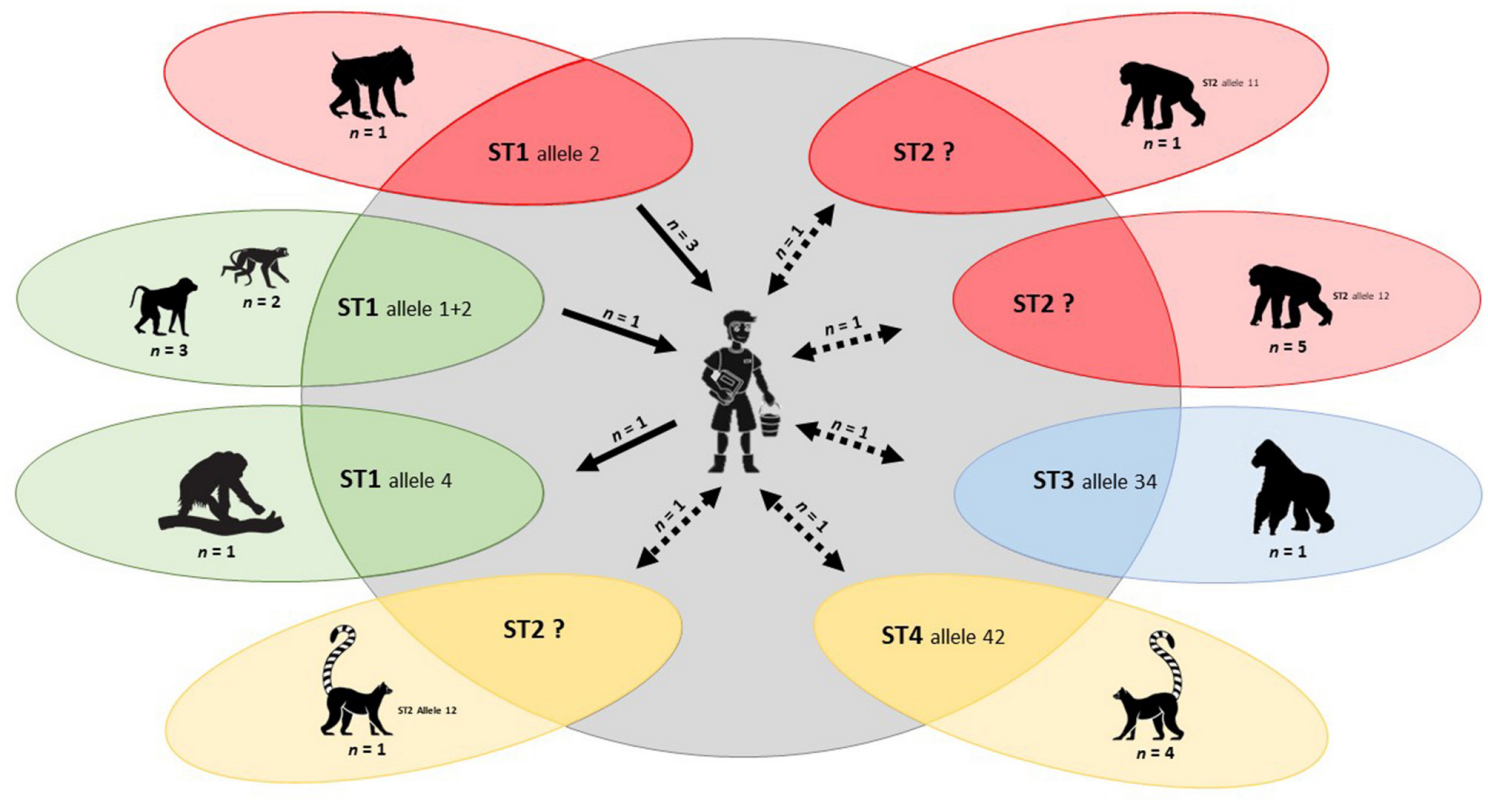
Molecular-based evidence of *Blastocystis* zoonotic transmission between captive NHP and their zookeepers in the present study. Individual zoological institutions where transmission events were detected are identified in different colours. Solid arrows represent zoonotic transmission events for which directionality (human-to-animal or animal-to-human) is well-established. Discontinuous arrows represent zoonotic transmission events for which directionality is unclear.

We obtained strong evidence of zoonotic transmission for *Blastocystis* sp. At the BZ, we identified ST3 allele 34 in a gorilla (*Gorilla gorilla*) and a zookeeper (Table 4; Figure 3).

At Faunia, we confirmed ST4 allele 42 in four lemurs (*Lemur catta*) and a zookeeper during the first sampling campaign, and ST2 allele 12 in a lemur (*L. catta*) and a zookeeper (unknown allele) during the second sampling campaign (Table 4; Figure 3).

At the MZA, we identified ST1 allele 2 in a mandrill (*Mandrillus sphinx*) and three zookeepers (all of them additionally carrying ST1 allele 1) during the first sampling campaign. Furthermore, ST2 alleles 11 and 12 were found in one and five chimpanzees (*P. troglodytes*), respectively, and ST2 (unknown allele) in a zookeeper during the same sampling period (Table 4; Figure 3).

During the second sampling campaign at the SZ, we observed *Blastocystis* ST1 allele 4 in a Sumatran orangutan (*Pongo abelii*) and a zookeeper, whereas we confirmed ST1 alleles 1+2 were confirmed in two Eastern black and white colobus (*Colobus guereza*), three lion-tailed macaques (*Macaca Silenus*), and a zookeeper (Table 4; Figure 3).

### Risk Factors for Zoonotic Transmission

We summarise in Table 5 the main sociodemographic, behavioural, and occupational factors potentially associated with protist infection/carriage in those NHP handlers where zoonotic transmission was demonstrated (see section Molecular Evidence of Zoonotic Transmission). The median age was 38 years (range: 27–61 years). The male/female ratio was 1.0. Two out of 10 handlers declared having diarrhoea the week before sampling. All of them were food handlers and had regular contact with faecal material from NHP, and most of them (90%, 9/10) had also contact with faecal material from animals other than NHP. Contact with infants or relatives with diarrhoea were reported by 30% (3/10) and 20% (2/10) of the participants, respectively. Three NHP handlers had a recent travel record to countries within the European Union (20%. 2/10) or out of it (10%, 1/10). Overall, pet dogs and cats were owned by 60% (6/10) and 20% (2/10) of the participants, respectively. Tap water was the main drinking water source in all cases, whereas consumption of bottled water or water from public fountains were declared by 30% (3/10) and 40% (4/10) of the participants. Additionally, 40% (4/10) of them swam in pools regularly. All of them declared washing their hands or fresh produce before eating frequently or always.

**Table 5:** Sociodemographic, behavioural, and work-related factors potentially associated with protist zoonotic transmission in zookeepers in the present study.

	**Zoological institution**									
**Variable**	**Barcelona Zoo**	**Faunia**			**Madrid Zoo Aquarium**			**Santillana Zoo**		
Subject	Suj46	Suj8	Suj104	Suj12	Suj14	Suj15	Suj17	Suj25	Suj91	Suj94
Sampling campaign	1	1	2	1	1	1	1	1	2	2
Positive to	*Bl*. ST3	*Bl*. ST4	*Bl*. ST2	*Bl*. ST1	*C. hominis, Bl*. ST2	*Bl*. ST1	*C. hominis*	*Bl*. ST1	*Bl*. ST1	*Bl*. ST1
Sex	Male	Female	Male	Female	Male	Female	Male	Female	Female	Male
Age (yrs.)	56	38	39	35	NS	27	56	35	38	61
Diarrhoea in past week	Yes	No	No	Yes	No	No	No	No	No	No
Contact with faeces from NHP	Yes	Yes	Yes	Yes	Yes	Yes	Yes	Yes	Yes	Yes
Contact with faeces from animals	Yes	Yes	Yes	Yes	Yes	Yes	No	Yes	Yes	Yes
other than NHP									
Contact with animals with diarrhoea	Unknown	Unknown	Yes	Yes	Unknown	Yes	Unknown	Yes	Yes	Yes
Food handler	Yes	Yes	Yes	Yes	Yes	Yes	Yes	Yes	Yes	Yes
Contact with infants	No	No	No	Yes	No	No	No	No	Yes	Yes
Diarrhoea in family	No	No	No	Yes	No	No	No	No	Yes	No
Recent travel (country/region)	Yes (EU)	Yes (EU)	No	No	NS	Yes (Malaysia)	No	No	No	No
Contact with dogs	No	Yes	Yes	No	No	Yes	Yes	No	Yes	Yes
Contact with cats	No	Yes	No	No	No	Yes	No	No	No	No
Main drinking water source	Tap	Tap	Tap	Tap	Tap	Tap	Tap	Tap	Tap	Tap
Secondary drinking water source	Fountain	Fountain	NS	Bottled	None	Fountain	Bottled	Tap	Bottled	Fountain
Swimming in pools	Yes	Yes	No	No	No	No	No	Yes	Yes	No
Handwashing	Always	Frequently	Always	Always	Always	Always	Frequently	Frequently	Always	Always
Vegetable washing	Always	Frequently	Always	Always	Always	Always	Frequently	Frequently	Always	Frequently

*Bl, Blastocystis; EU, European Union; NS, not specified*.

## Discussion

This is one of the largest molecular-based epidemiological studies conducted in Europe to elucidate the occurrence, genetic diversity and zoonotic potential of intestinal protist species in captive NHP. The study was conducted in six different zoological institutions from France, Germany, and Spain and involved individuals from 35 different NHP genera and their zookeepers.

Several epidemiological surveys have demonstrated the association between *Cryptosporidium* infection and sporadic cases or outbreaks of diarrhoea in captive NHP (4, 40, 41). The parasite has also been linked to decrease microbial diversity and increase community dissimilarity in the gut microbiome of susceptible NHP hosts (42). *Cryptosporidium* infection rates have been typically reported in the range of 3–6% in NHP species including baboons, gibbons, macaques, and orangutans (41, 43–48). Other surveys failed to detect the parasite in their captive NHP collections (13, 29, 49–53). This variability is clearly reflected in the present study, where an overall *Cryptosporidium* infection rate of 1.0% was found, but the parasite was only detected in two of the six participating zoological institutions. A long-term study conducted at the Barcelona Zoo in Spain suggested that persistent *Cryptosporidium* infections (probably associated to immunosuppression by captivity-induced stress) can be difficult to control and eradicate (54). Regarding molecular diversity, captive NHP primates have been shown to be infected by at least four *Cryptosporidium* species including *C. hominis, C. parvum, C. muris*, and *C. andersoni* (4, 41, 44–47, 49). Of them, *C. hominis* and *C. parvum* are the species most consistently found, including the present study. When successful, *gp60* family sub-genotype analyses have revealed the presence of IdA14 in laboratory macaques (45), IId A15G2R1 in a rhesus macaque (46), and IaA20R3a, IoA17a, IoA17b, and IiA17 in long-tailed macaques (41). Unfortunately, none of the *C. hominis* and *C. parvum* isolates identified here could be genotyped at the *gp60* locus, very likely because of insufficient starting amount of parasitic DNA in the faecal samples. This fact may be indicative of light infections, compatible with the apparent absence of symptoms in the surveyed NHP.

Captive NHP are suitable hosts for several species of the *Entamoeba* genus including *E. bangladeshi, E. chattoni, E. coli, E. dispar, E. ecuadoriensis, E. hartmanni, E. histolytica, E. moshkovskii, E. nutalli*, and *E. polecki* (8, 55). Because all these species are morphologically indistinguishable and their strains show different virulence capabilities (15), a correct differential diagnosis is important. Among them, *E. histolytica* is regarded as the most relevant species given its zoonotic potential and veterinary public health impact (56). Subclinical *E. histolytica* infections have been frequently reported in captive NHP at rates varying from 16% to 40% globally (8, 55, 57–59). Fatal amebiasis cases affecting spider monkeys, mantled guerezas, and Hanuman langurs have also been sporadically reported (60, 61). Nonetheless, other surveys carried out in European (13, 51), African (29), and Asian (62, 63) countries did not find *E. histolytica* in the captive NHP populations investigated. This is also the case in the present study, were *E. histolytica* was undetected in all six zoological institutions under investigation. In contrast, an overall occurrence rate of 8.5% was observed for non-pathogenic *E. dispar*.

*Giardia duodenalis* is a common finding in faecal samples of captive NHP globally. Only in European zoological institutions, reported infection rates were in the range of 6–70% in Belgium (64), Croatia (65), Italy (66), Slovakia (67), and Spain (13, 68), although the parasite was apparently absent in other surveys conducted in Italy (50) and Norway (49). All the above-mentioned studies were carried out in asymptomatic NHP. Remarkably, very few surveys have demonstrated an association between the presence of *G. duodenalis* infection and the occurrence of clinical manifestations (69), strongly suggesting that the pathogenic role of *G. duodenalis* in captive NHP is limited. This is also the case of the present study, where *G. duodenalis* was identified in all six zoological institutions investigated at an overall infection rate of 15.8% and all infected NHP were apparently healthy animals. Our genetic analyses revealed that eight out of 10 *G. duodenalis* infections were caused by the assemblage B of the parasite, and that all the genetic variants detected (sub-assemblages AI, AII, and BIV) were zoonotic (70). This predominance of assemblage B over assemblage A has been confirmed in other NHP populations from Belgium and the Netherlands (71), China (72–74), Croatia (65), Italy (66), the Republic of Congo (49), Slovakia (67), Sweden (75), and Thailand (52). However, it should be noted that this trend is far from general, and the opposite result (preponderance of assemblage A over assemblage B) has also been reported in captive NHP belonging to the genera *Alouatta* and *Ateles* in Brazil (76, 77) and in different NHP collections from Spanish zoological gardens (13, 68). Mixed assemblage A+B infections seem also frequent in some settings (71). Infections by *G. duodenalis* assemblages other than A or B are less frequent but not rare. For instance, assemblage E has been described circulating in rhesus macaques and squirrel monkeys in China (46).

*Blastocystis* sp. is regarded as the most common eukaryotic parasite present in human faecal samples (78). This protist has been implicated in the aetiology of a range of different intestinal (i.e., irritable bowel syndrome, diarrhoea) and extra-intestinal (i.e., urticarial) diseases (79), but its true clinical significance remains unclear and controversial because asymptomatic carriage is the norm rather than the exception. This very same statement is also valid for captive NHP, where infection/carriage rates over 20% and up to 100% have been consistently found in studies conducted in Bangladesh (80), Brazil (81, 82), China (83), France (53), Italy (50), Peru (84), Republic of the Congo and Senegal (53), and Spain (13, 43). The occurrence rates identified in the present study (2–41%) are well in the range of those reported above, corroborating *Blastocystis* sp. as the overall most prevalent protist species detected in the surveyed NHP population. In a seminal large genotyping study conducted in both captive and wild NHP, subtypes ST1–ST3 accounted for 83% of the *Blastocystis* isolates identified, followed by ST8, ST5, ST4, ST13, and ST15 (85). Interestingly, ST1–ST3 distribution was independent of NHP group or geographical association, ST5 was seen only in apes and old world monkeys, and ST8 primarily in species native to Asia or South America (85). Subsequent surveys have confirmed (13, 53, 82, 84) or extended these preliminary molecular data. For instance, ST7 has been described in cynomolgus monkeys in Italy (50), ST13 in langurs and golden snub-nosed monkeys in Bangladesh (80) and China (83), ST17 in squirrel monkeys in China (86), and ST19 in a rhesus macaque in the latter country (83). In line with these results, six *Blastocystis* subtypes (ST1–ST5, ST8, and ST13) were identified in the present study, with ST1 being the most prevalent one at the global level. Of note, ST1 was only observed in Spanish (but not French or German) zoological institutions. At present, we do not have a clear explanation for this finding, but the low number of *Blastocystis* isolates genotyped in the French and German collections may have biassed, at least partially, these results.

In humans, *E. bieneusi* is an opportunistic pathogen primarily infecting immunocompromised individuals (7). However, the veterinary health implications of this microsporidia in captive NHP remains largely unknown. Earlier studies associated *E. bieneusi* infection with hepatobiliary and intestinal disease and proliferative serositis in simian immunodeficiency virus-infected macaques (87, 88). Available epidemiological data seem to indicate that *E. bieneusi* is a relatively common finding in African captive NHP including resident chimpanzees in sanctuaries from Cameroon and Kenya (89) and recently captured olive baboons in the latter country (44). Reported infection rates ranged from 1–12% and involved genotypes A, D, EbpA, KB-1 to KB-6, PigEBITS5, Peru7, and Peru11. Much higher infection rates and genetic diversity frequencies have been documented in NHP collections from zoological gardens in Asian countries, particularly in China. In that country, *E. bieneusi* infection rates varied from 11–46% (46, 90–93). All these studies included sample panels over 150 animals. The highest infection rates were detected in golden snub-nosed monkeys (46%) (92), and long-tailed macaques (31%) (93). Remarkably, an extensive genetic heterogeneity within *E. bieneusi* was found in these studies, with the description of 5–25 distinct genotypes (including many novel genetic variants) circulating in the NHP collections investigated. Genotypes D, BEB6, CM1, CM4, O, EbpC, J, and Type IV were more prevalently found. An *E. bieneusi* occurrence rate of 27% has also been reported in captive NHP in Bangladesh, with genotype D accounting for most (82%) of the infections (94). Comparatively, much less epidemiological and molecular information is available from NHP collections in European countries, where the parasite is typically absent or undetected (13, 43, 89). In the only *E. bieneusi* isolate characterised to date, genotype D has been identified in a resident chimpanzee at the Bratislava zoo in Slovakia (89). Data presented here confirm that Microsporidia infections by *E. bieneusi* occurs only sporadically in European captive NHP. Of particular interest was the finding of genotype CM18 infecting a black-capped squirrel monkey and a red-handed tamarin in France, and a Western lowland gorilla in Spain. This specific genotype was only detected previously in a ring-tailed lemur in China and, based on phylogenetic analyses, forms part of the genetic Group 9 that includes *E. bieneusi* genetic variants with strong preference to NHP hosts (91).

The zoonotic ciliate *B. coli* is a common parasite of swine, but also of other mammal species including NHP (25). Although asymptomatic commensalism predominates in NHP, invasion of the colonic mucosa can lead to diarrhoea and dysentery and set the stage for local or systemic spread (5, 69, 95). In addition, the presence and abundance of *B. coli* has been linked with the quality of lactation milk samples in captive rhesus macaques (96). Infections by *B. coli* in captive NHP have been reported at rates of 13% in Belgium (64), of 11% in Brazil (48), of 22% in Italy (50), and of 4% in Spain (13). In a large multicentre study involving 11 zoological institutions in Europe and two sanctuaries in Africa, an overall *B. coli* occurrence rate of 50% was found in great apes including chimpanzees, bonobos, and gorillas. Remarkably, in that very same survey wild NHP were uninfected by the parasite (97). In the present study, *B. coli* was identified at an overall low (1.7%) rate and only in one of the six participating zoological institutions. It has been suggested that starch-rich diet (such as those typically present at zoos) might be responsible for high intensities of infection of *B. coli* in captive NHP (98).

The entodiniomorphid ciliate *T. abrassarti* is a colonic mutualist of captive and wild African great apes including chimpanzees, bonobos, and gorillas (26). Prevalence rates near 100% have been documented in wild chimpanzees in Uganda (99). In captive NHP populations, *T. abrassarti* has been identified by microscopy examination of freshly collected faecal samples at rates of 7% in chimpanzees in Gabon (100), and of 17–100% in European zoological institutions from Czech Republic, France, Germany, Ireland, the Netherlands, Slovak Republic, and Switzerland (101). In the latter survey, *T. abrassarti* was also found in 50–60% of captive bonobos in Belgium and Germany (101). Additionally, this ciliate is also known to be present in captive gorillas in the Czech Republic and UK (9). Remarkably, much lower occurrence rates (range: 0–13%) have been reported in captive NHP in PCR-based studies conducted in Spain (13) Côte d'Ivoire and Peru (29). *Troglodytella abrassarti* does not form cysts and decomposition of trophozoites began immediately after defecation (102). This means that if DNA extraction is not conducted in fresh faecal samples, the obtained genomic material might be of suboptimal quality for the detection of *T. abrassarti*. This fact would also explain the low occurrence rate (1.5%) found in this study, were most NHP faecal samples were kept frozen for several weeks before processing.

Perhaps the main contributions of the present survey was the demonstration of *Blastocystis* zoonotic transmission between captive NHP and their zookeepers in different Spanish zoological institutions. Because allele 4 is the dominant ST1 genetic variant circulating in the Spanish human population (103, 104) and alleles 1 and 2 in captive NHP (13, present study), the finding of ST1 allele 4 in an orangutan and a zookeeper in SZ was interpreted as a human-to-animal transmission event. In contrast, an animal-to-human transmission can be inferred from the finding of ST1 alleles 1 + 2 in colobus and macaque monkeys and a zookeeper at the same zoological institution, and from the finding of ST1 allele 2 in a mandrill and three zookeepers at MZA. Evidence of zoonotic transmission events was also gained from the finding of ST2 alleles 11 or 12 in a lemur at Faunia and chimpanzees at MZA and their respective zookeepers, although the exact alleles of the *Blastocystis* human isolates could not be determined. Furthermore, ST3 allele 34 was identified in a gorilla and a zookeeper at BZ, and ST4 allele 42 in lemurs and a zookeeper at Faunia. Because of lack of intra-subtype molecular data within ST2, ST3, and ST4 of NHP origin, the directionality of these zoonotic events remains to be fully elucidated. Of note, zoonotic transmission of *Blastocystis* ST1 and ST8 between captive NHP and their zookeepers has been previously documented in zoological gardens in Spain and UK, respectively (12, 13). Similarly, *C. hominis* zoonotic transmission was highly suspected between a chimpanzee and two zookeepers at MZA, although lack of genotyping data at the *gp60* locus precluded us to ascertain the extent and directionality of this event.

In conclusion, this molecular-based survey revealed that a high proportion of the captive NHP (41%) and their zookeepers (30%) investigated were infected/colonised by intestinal protist species of potential or uncertain pathogenicity. Besides *Blastocystis* sp. (21%), *G. duodenalis* (16%), and *E. dispar* (7%), all the remaining protist species were identified at low ( ≤ 2%) rates in the human and NHP populations under study, whereas *E. histolytica* was apparently absent. Large variations in the occurrence rates were found according to NHP host species considered, sample size, sampling period, and zoological institution investigated. These differences may be attributed, at least partially, to intrinsic biological differences among resident NHP collections in each participating zoological institution, environmental (including climatic) conditions, and handling and management practises. Remarkably, strong evidence of zoonotic transmission animal-to-human and human-to-animal was gathered for *Blastocystis* sp. (the predominant protist species in both humans and NHP), and, to a lesser extent, for *C. hominis*. Molecular-based studies constitute a powerful tool for the monitoring of intestinal protist species in captive NHP and their zookeepers, assisting in the identification of clinical cases and sources of infection, and assessing potential transmission risk to (or from) other resident animals and visitors.

## Data Availability Statement

The datasets presented in this study can be found in online repositories. The names of the repository/repositories and accession number(s) can be found in the article/Supplementary Material.

## Ethics Statement

This study was approved by Ethics Committee of the Health Institute Carlos III under the reference number CEI PI 90_2018-v2. The patients/participants provided their written informed consent to participate in this study. Written informed consent was obtained from the individual(s) for the publication of any potentially identifiable images or data included in this article.

## Author Contributions

PK, DG-B, FP-G, RC-B, and DC conceived and designed the study, analysed the data, and contributed to writing the manuscript. PK, EM-N, AG, MA-P, HF-B, MR-F, BM, J-PG, TK-W, AW, AD, BB, EI, and AM, carried out the study. All authors contributed to the article and approved the submitted version.

## Funding

This study was funded by the Health Institute Carlos III (ISCIII), Spanish Ministry of Economy and Competitiveness under project PI16CIII/00024. DG-B was recipient of a Sara Borrell Postdoctoral Fellowship (CD19CIII/00011) funded by the Spanish Ministry of Science, Innovation and Universities. AD was recipient of a PFIS contract (FI20CIII/00002) funded by the Spanish Ministry of Science and Innovation and Universities.

## Conflict of Interest

The authors declare that the research was conducted in the absence of any commercial or financial relationships that could be construed as a potential conflict of interest.

## Publisher's Note

All claims expressed in this article are solely those of the authors and do not necessarily represent those of their affiliated organizations, or those of the publisher, the editors and the reviewers. Any product that may be evaluated in this article, or claim that may be made by its manufacturer, is not guaranteed or endorsed by the publisher.

## References

[1] Levecke B. The Importance of Gastrointestinal Protozoa in Captive Non-Human Primates. [dissertation thesis]. Ghent: Ghent University (2010).

[2] Verweij JJ Vermeer J Brienen EA Blotkamp C Laeijendecker D van Lieshout L. *Entamoeba histolytica infections* in captive primates. Parasitol Res. (2003) 90:100–103. 10.1007/s00436-002-0808-z 12756542

[3] Kramer JA Hachey AM Wachtman LM Mansfield KG. Treatment of giardiasis in common marmosets (*Callithrix jacchus*) with tinidazole. Comp Med. (2009) 59:174–179. 19389310 PMC2703147

[4] da Silva AJ Cacciò S Williams C Won KY Nace EK Whittier C. Molecular and morphologic characterization of a *Cryptosporidium* genotype identified in lemurs. Vet Parasitol. (2003) 111:297–307. 10.1016/S0304-4017(02)00384-9 12559709

[5] Lankester F Mätz-Rensing K Kiyang J Jensen SA Weiss S Leendertz FH. Fatal ulcerative colitis in a western lowland gorilla (*Gorilla gorilla gorilla*). J Med Primatol. (2008) 37:297–302. 10.1111/j.1600-0684.2008.00287.x 18466283

[6] Kumarasamy V Anbazhagan D Subramaniyan V Vellasamy S. *Blastocystis sp*., parasite associated with gastrointestinal disorders: An overview of its pathogenesis, immune modulation and therapeutic strategies. Curr Pharm Des. (2018) 24:3172–5. 10.2174/1381612824666180807101536 30084327

[7] Li W Xiao L. Ecological and public health significance of *Enterocytozoon bieneusi*. One Health. (2020) 12:100209. 10.1016/j.onehlt.2020.100209 33426263 PMC7779778

[8] Levecke B Dreesen L Dorny P Verweij JJ Vercammen F Casaert S. Molecular identification of *Entamoeba* spp. in captive nonhuman primates. J Clin Microbiol. (2010) 48:2988–90. 10.1128/JCM.00013-10 20573870 PMC2916622

[9] Modrý D Petrzelková KJ Pomajbíková K Tokiwa T Krízek J Imai S. The occurrence and ape-to-ape transmission of the entodiniomorphid ciliate *Troglodytella abrassarti* in captive gorillas. J Eukaryot Microbiol. (2009) 56:83–87. 10.1111/j.1550-7408.2008.00369.x 19335778

[10] de Waal T. Advances in diagnosis of protozoan diseases. Vet Parasitol. (2012) 189:65–74. 10.1016/j.vetpar.2012.03.033 22503386

[11] Meurs L Polderman AM Vinkeles Melchers NV Brienen EA Verweij JJ. Diagnosing polyparasitism in a high-prevalence setting in Beira, mozambique: detection of intestinal parasites in fecal samples by microscopy and real-time PCR. PLoS Negl Trop Dis. (2017) 11:e0005310. 10.1371/journal.pntd.0005310 28114314 PMC5289637

[12] Stensvold CR Alfellani MA Nørskov-Lauritsen S Prip K Victory EL Maddox C. Subtype distribution of *Blastocystis* isolates from synanthropic and zoo animals and identification of a new subtype. Int J Parasitol. (2009) 39:473–9. 10.1016/j.ijpara.2008.07.006 18755193

[13] Köster PC Dashti A Bailo B Muadica AS Maloney JG Santín M. Occurrence and genetic diversity of protist parasites in captive non-human primates, zookeepers, and free-living sympatric rats in the Córdoba zoo conservation centre, southern Spain. Animals. (2021) 11:700. 10.3390/ani11030700 33807707 PMC8035673

[14] Weedall GD Clark CG Koldkjaer P Kay S Bruchhaus I Tannich E. Genomic diversity of the human intestinal parasite *Entamoeba histolytica*. Genome Biol. (2012) 13:R38. 10.1186/gb-2012-13-5-r38 22630046 PMC3446291

[15] Das K Sardar SK Ghosal A Saito-Nakano Y Dutta S Nozaki T. Multilocus sequence typing (MLST) of *Entamoeba histolytica* identifies *kerp2* as a genetic marker associated with disease outcomes. Parasitol Int. (2021) 83:102370. 10.1016/j.parint.2021.102370 33932601

[16] Cai W Ryan U Xiao L Feng Y. Zoonotic giardiasis: an update. Parasitol Res. (2021) 120:4199–4218. 10.1007/s00436-021-07325-2 34623485

[17] Messa A Jr Köster PC Garrine M Gilchrist C Bartelt LA Nhampossa T. Molecular diversity of *Giardia duodenalis* in children under 5 years from the Manhiça district, Southern Mozambique enrolled in a matched case-control study on the aetiology of diarrhoea. PLoS Negl Trop Dis. (2021) 15:e0008987. 10.1371/journal.pntd.0008987 33465074 PMC7846004

[18] Zahedi A Bolland SJ Oskam CL Ryan U. Cryptosporidium abrahamseni n. sp. (Apicomplexa: Cryptosporidiiae) from red-eye tetra (Moenkhausia sanctaefilomenae). Exp Parasitol. (2021) 223:108089. 10.1016/j.exppara.2021.108089 33639135

[19] Feng Y Ryan UM Xiao L. Genetic diversity and population structure of *Cryptosporidium*. Trends Parasitol. (2018) 34:997–1011. 10.1016/j.pt.2018.07.009 30108020

[20] Widmer G Köster PC Carmena D. Cryptosporidium hominis infections in non-human animal species: revisiting the concept of host specificity. Int J Parasitol. (2020) 50:253–62. 10.1016/j.ijpara.2020.01.005 32205089

[21] Maloney JG Santin M. Mind the gap: new full-length sequences of *Blastocystis* subtypes generated via Oxford Nanopore Minion sequencing allow for comparisons between full-length and partial sequences of the small subunit of the ribosomal RNA gene. Microorganisms. (2021) 9:997. 10.3390/microorganisms9050997 34063045 PMC8147991

[22] Higuera A Herrera G Jimenez P García-Corredor D Pulido-Medellín M Bulla-Castañeda DM. Identification of multiple *Blastocystis* subtypes in domestic animals from Colombia using amplicon-based next generation sequencing. Front Vet Sci. (2021) 8:732129. 10.3389/fvets.2021.732129 34504891 PMC8421793

[23] Hublin JSY Maloney JG Santin M. *Blastocystis* in domesticated and wild mammals and birds. Res Vet Sci. (2021) 135:260–82. 10.1016/j.rvsc.2020.09.031 33046256

[24] Li W Feng Y Santin M. Host specificity of *Enterocytozoon bieneusi* and public health implications. Trends Parasitol. (2019) 35:436–51. 10.1016/j.pt.2019.04.004 31076351

[25] Ponce-Gordo F García-Rodríguez JJ. Balantioides coli. Res Vet Sci. (2021) 35:424–31. 10.1016/j.rvsc.2020.10.028 33183780

[26] Vallo P PetrŽelková KJ Profousová I Petrášová J Pomajbíková K Leendertz F. Molecular diversity of entodiniomorphid ciliate *Troglodytella abrassarti* and its coevolution with chimpanzees. Am J Phys Anthropol. (2012) 148:525–33. 10.1002/ajpa.22067 22576323

[27] Köster PC Renelies-Hamilton J Dotras L Llana M Vinagre-Izquierdo C Prakas P. Molecular detection and characterization of intestinal and blood parasites in wild chimpanzees (*Pan troglodytes verus*) in Senegal. Animals. (2021) 11:3291. 10.3390/ani11113291 34828022 PMC8614354

[28] Köster PC Lapuente J Dashti A Bailo B Muadica AS González-Barrio D. Enteric protists in critically endangered wild chimpanzees (*Pan troglodytes verus*) and humans in the Comoé National Park, Côte d'Ivoire. Primates. (2021). 10.1007/s10329-021-00963-1 34997384

[29] Köster PC Lapuente J Pizarro A Prieto-Pérez L Pérez-Tanoira R Dashti A. Presence and genetic diversity of enteric protists in captive and semi-captive non-human primates in Côte d'Ivoire, Sierra Leone, and Peru. Int J Parasitol Parasites Wildl. (2021) 17:26–34. 10.1016/j.ijppaw.2021.12.004 34976722 PMC8688894

[30] Tiangtip R Jongwutiwes S. Molecular analysis of *Cryptosporidium* species isolated from HIV-infected patients in Thailand. Trop Med Int Health. (2002) 7:357–64. 10.1046/j.1365-3156.2002.00855.x 11952952

[31] Verweij JJ Oostvogel F Brienen EA Nang-Beifubah A Ziem J Polderman AM. Short communication: prevalence of *Entamoeba histolytica* and *Entamoeba dispar* in northern Ghana. Trop Med Int Health. (2003) 8:1153–6. 10.1046/j.1360-2276.2003.01145.x 14641852

[32] Gutiérrez-Cisneros MJ Cogollos R López-Vélez R Martín-Rabadán P Martínez-Ruiz R Subirats M. Application of real-time PCR for the differentiation of *Entamoeba histolytica* and *E*. dispar in cyst-positive faecal samples from 130 immigrants living in Spain. Ann Trop Med Parasitol. (2010) 104:145–9. 10.1179/136485910X12607012373759 20406581

[33] Verweij JJ Schinkel J Laeijendecker D van Rooyen MA van Lieshout L Polderman AM. Real-time PCR for the detection of *Giardia lamblia*. Mol Cell Probes. (2003) 17:223–5. 10.1016/S0890-8508(03)00057-4 14580396

[34] Read CM Monis PT Thompson RC. Discrimination of all genotypes of *Giardia duodenalis* at the glutamate dehydrogenase locus using PCR-RFLP. Infect Genet Evol. (2004) 4:125–30. 10.1016/j.meegid.2004.02.001 15157630

[35] Lalle M Pozio E Capelli G Bruschi F Crotti D Cacciò SM. Genetic heterogeneity at the beta-giardin locus among human and animal isolates of *Giardia duodenalis* and identification of potentially zoonotic subgenotypes. Int J Parasitol. (2005) 35:207–13. 10.1016/j.ijpara.2004.10.022 15710441

[36] Sulaiman IM Fayer R Bern C Gilman RH Trout JM Schantz PM. Triosephosphate isomerase gene characterization and potential zoonotic transmission of *Giardia duodenalis*. Emerg Infect Dis. (2003) 9:1444–52. 10.3201/eid0911.030084 14718089 PMC3035538

[37] Scicluna SM Tawari B Clark CG. DNA barcoding of *Blastocystis*. Protist. (2006) 157:77–85. 10.1016/j.protis.2005.12.001 16431158

[38] Buckholt MA Lee JH Tzipori S. Prevalence of *Enterocytozoon bieneusi* in swine: an 18-month survey at a slaughterhouse in massachusetts. Appl Environ Microbiol. (2002) 68:2595–9. 10.1128/AEM.68.5.2595-2599.2002 11976142 PMC127518

[39] Ponce-Gordo F Fonseca-Salamanca F Martínez-Díaz RA. Genetic heterogeneity in internal transcribed spacer genes of *Balantidium coli* (Litostomatea, Ciliophora). Protist. (2011) 162:774–94. 10.1016/j.protis.2011.06.008 21840258

[40] Charles-Smith LE Cowen P Schopler R. Environmental and physiological factors contributing to outbreaks of *Cryptosporidium* in Coquerel's sifaka (*Propithecus coquereli*) at the Duke Lemur center: 1999-2007. J Zoo Wildl Med. (2010) 41:438–44. 10.1638/2009-0160.1 20945641

[41] Zhao W Zhou H Jin H Liu M Qiu M Li L. Molecular prevalence and subtyping of *Cryptosporidium hominis* among captive long-tailed macaques (*Macaca fascicularis*) and rhesus macaques (*Macaca mulatta*) from Hainan Island, southern China. Parasit Vect. (2019) 12:192. 10.1186/s13071-019-3449-0 31039801 PMC6492332

[42] McKenney EA Greene LK Drea CM Yoder AD. Down for the count: *Cryptosporidium* infection depletes the gut microbiome in Coquerel's sifakas. Microb Ecol Health Dis. (2017) 28:1335165. 10.1080/16512235.2017.1335165 28740461 PMC5508644

[43] Pérez Cordón G Hitos Prados A Romero D Sánchez Moreno M Pontes A Osuna A. Intestinal parasitism in the animals of the zoological garden “Peña Escrita” (Almuñecar, Spain). Vet Parasitol. (2008) 156:302–309. 10.1016/j.vetpar.2008.05.023 18639383

[44] Li W Kiulia NM Mwenda JM Nyachieo A Taylor MB Zhang X. *Cyclospora papionis, Cryptosporidium hominis*, and human-pathogenic *Enterocytozoon bieneusi* in captive baboons in Kenya. J Clin Microbiol. (2011) 49:4326–9. 10.1128/JCM.05051-11 21956988 PMC3232936

[45] Ye J Xiao L Li J Huang W Amer SE Guo Y. Occurrence of human-pathogenic *Enterocytozoon bieneusi, Giardia duodenalis* and *Cryptosporidium* genotypes in laboratory macaques in Guangxi, China. Parasitol Int. (2014) 63:132–7. 10.1016/j.parint.2013.10.007 24157444

[46] Du SZ Zhao GH Shao JF Fang YQ Tian GR Zhang LX. *Cryptosporidium* spp., *Giardia intestinalis*, and *Enterocytozoon bieneusi* in captive non-human primates in Qinling mountains. Korean J Parasitol. (2015) 53:395–402. 10.3347/kjp.2015.53.4.395 26323837 PMC4566506

[47] Mynárová A Foitová I Kváč M Květonová D Rost M Morrogh-Bernard H. Prevalence of *Cryptosporidium* spp., *Enterocytozoon bieneusi, Encephalitozoon* spp. and Giardia intestinalis in wild, semi-wild and captive orangutans (Pongo abelii and Pongo pygmaeus) on Sumatra and Borneo, Indonesia. PLoS ONE. (2016) 11:e0152771. 10.1371/journal.pone.0152771 27031241 PMC4816420

[48] Barbosa ADS Pinheiro JL Dos Santos CR de Lima CSCC Dib LV Echarte GV. Gastrointestinal parasites in captive animals at the Rio de Janeiro Zoo. Acta Parasitol. (2020) 65:237–49. 10.2478/s11686-019-00145-6 31960215

[49] Debenham JJ Atencia R Midtgaard F Robertson LJ. Occurrence of *Giardia* and *Cryptosporidium* in captive chimpanzees (*Pan troglodytes*), mandrills (*Mandrillus sphinx*) and wild Zanzibar red colobus monkeys (*Procolobus kirkii*). J Med Primatol. (2015) 44:60–5. 10.1111/jmp.12158 25612000

[50] Zanzani SA Gazzonis AL Epis S Manfredi MT. Study of the gastrointestinal parasitic fauna of captive non-human primates (*Macaca fascicularis*). Parasitol Res. (2016) 115:307–12. 10.1007/s00436-015-4748-9 26374536

[51] Osman M El Safadi D Benamrouz-Vanneste S Cian A Moriniere R Gantois N. Prevalence, transmission, and host specificity of *Cryptosporidium* spp. in various animal groups from two French zoos. Parasitol Res. (2017) 116:3419–22. 10.1007/s00436-017-5645-1 29030716

[52] Tangtrongsup S Sripakdee D Malaivijitnond S Angkuratipakorn R Lappin M. Intestinal parasites and the occurrence of zoonotic *Giardia duodenalis* genotype in captive gibbons at Krabokkoo Wildlife Breeding Center, Thailand. Front Vet Sci. (2019) 6:110. 10.3389/fvets.2019.00110 31106211 PMC6499157

[53] Menu E Davoust B Mediannikov O Akiana J Mulot B Diatta G. Occurrence of ten protozoan enteric pathogens in three non-human primate populations. Pathogens. (2021) 10:280. 10.3390/pathogens10030280 33801236 PMC8001678

[54] Gracenea M Gómez MS Torres J Carné E Fernández-Morán J. Transmission dynamics of *Cryptosporidium* in primates and herbivores at the Barcelona zoo: a long-term study. Vet Parasitol. (2002) 104:19–26. 10.1016/S0304-4017(01)00611-2 11779652

[55] Regan CS Yon L Hossain M Elsheikha HM. Prevalence of *Entamoeba* species in captive primates in zoological gardens in the UK. PeerJ. (2014) 2:e492. 10.7717/peerj.492 25097822 PMC4121542

[56] Li J Cui Z Li X Zhang L. Review of zoonotic amebiasis: Epidemiology, clinical signs, diagnosis, treatment, prevention and control. Res Vet Sci. (2021) 136:174–81. 10.1016/j.rvsc.2021.02.021 33676155

[57] Smith JM Meerovitch E. Primates as a source of *Entamoeba histolytica*, their zymodeme status and zoonotic potential. J Parasitol. (1985) 71:751–6. 10.2307/3281708 2869116

[58] Munene E Otsyula M Mbaabu DA Mutahi WT Muriuki SM Muchemi GM. Helminth and protozoan gastrointestinal tract parasites in captive and wild-trapped African non-human primates. Vet Parasitol. (1998) 78:195–201. 10.1016/S0304-4017(98)00143-5 9760061

[59] Rivera WL Yason JA Adao DE. *Entamoeba histolytica* and *E*. dispar infections in captive macaques (Macaca fascicularis) in the Philippines. Primates. (2010) 51:69–74. 10.1007/s10329-009-0174-x 19862480

[60] Márquez-Monter H Fuentes-Orozco R Correa-Lemus I Becker I. Invasive amebiasis in a spider monkey (*Ateles geoffroyi*). Case report and a short review of the literature of amebiasis in non-human primates. Arch Invest Med. (1991) 22:75–8. 1819979

[61] Ulrich R Böer M Herder V Spitzbarth I Hewicker-Trautwein M Baumgärtner W. Epizootic fatal amebiasis in an outdoor group of old world monkeys. J Med Primatol. (2010) 39:160–5. 10.1111/j.1600-0684.2010.00405.x 20202078

[62] Tachibana H Cheng XJ Kobayashi S Matsubayashi N Gotoh S Matsubayashi K. High prevalence of infection with *Entamoeba dispar*, but not *E* histolytica, in captive macaques. Parasitol Res. (2001) 87:14–7. 10.1007/s004360000289 11199843

[63] Feng M Yang B Yang L Fu Y Zhuang Y Liang L. High prevalence of *Entamoeba* infections in captive long-tailed macaques in China. Parasitol Res. (2011) 109:1093–7. 10.1007/s00436-011-2351-2 21484347

[64] Levecke B Dorny P Geurden T Vercammen F Vercruysse J. Gastrointestinal protozoa in non-human primates of four zoological gardens in Belgium. Vet Parasitol. (2007) 148:236–46. 10.1016/j.vetpar.2007.06.020 17656023

[65] Beck R Sprong H Bata I Lucinger S Pozio E Cacciò SM. Prevalence and molecular typing of Giardia spp. in captive mammals at the zoo of Zagreb, Croatia. Vet Parasitol. (2011) 175:40–6. 10.1016/j.vetpar.2010.09.026 20970259

[66] Berrilli F Prisco C Friedrich KG Di Cerbo P Di Cave D De Liberato C. *Giardia duodenalis* assemblages and *Entamoeba* species infecting non-human primates in an Italian zoological garden: zoonotic potential and management traits. Parasit Vect. (2011) 4:199. 10.1186/1756-3305-4-199 21988762 PMC3214166

[67] Mravcová K Štrkolcová G Mucha R Goldová M. Zoonotic assemblages of *Giardia duodenalis* in captive non-human primates from the largest zoo in Slovakia. J Parasit Dis. (2021) 45:302–5. 10.1007/s12639-020-01324-3 34295025 PMC8254674

[68] Martínez-Díaz RA Sansano-Maestre J Martínez-Herrero MC Ponce-Gordo F Gómez-Muñoz MT. Occurrence and genetic characterization of *Giardia duodenalis* from captive nonhuman primates by multi-locus sequence analysis. Parasitol Res. (2011) 109:539–44. 10.1007/s00436-011-2281-z 21327988

[69] Sestak K Merritt CK Borda J Saylor E Schwamberger SR Cogswell F. Infectious agent and immune response characteristics of chronic enterocolitis in captive rhesus macaques. Infect Immun. (2003) 71:4079–86. 10.1128/IAI.71.7.4079-4086.2003 12819098 PMC162015

[70] Sprong H Cacciò SM van der Giessen JW. ZOOPNET network and partners. Identification of zoonotic genotypes of *Giardia duodenalis*. PLoS Negl Trop Dis. (2009) 3:e558. 10.1371/journal.pntd.0000558 19956662 PMC2777335

[71] Levecke B Geldhof P Claerebout E Dorny P Vercammen F Cacciò SM. Molecular characterisation of *Giardia duodenalis* in captive non-human primates reveals mixed assemblage A and B infections and novel polymorphisms. Int J Parasitol. (2009) 39:1595–601. 10.1016/j.ijpara.2009.05.013 19523472

[72] Zhong Z Tian Y Li W Huang X Deng L Cao S. Multilocus genotyping of *Giardia duodenalis* in captive non-human primates in Sichuan and Guizhou provinces, Southwestern China. PLoS ONE. (2017) 12:e0184913. 10.1371/journal.pone.0184913 28910395 PMC5599030

[73] Zhang X Wang L Lan X Dan J Ren Z Cao S. Occurrence and multilocus genotyping of *Giardia duodenalis* in captive non-human primates from 12 zoos in China. PLoS ONE. (2020) 15:e0228673. 10.1371/journal.pone.0228673 32017796 PMC6999901

[74] Liu H Wang B Yin J Yuan Z Jiang Y Zhang J. Investigation of giardiasis in captive animals in zoological gardens with strain typing of assemblages in China. Parasitology. (2021) 148:1360–5. 10.1017/S0031182021000913 34100347 PMC11010148

[75] Lebbad M Mattsson JG Christensson B Ljungström B Backhans A Andersson JO. From mouse to moose: multilocus genotyping of *Giardia* isolates from various animal species. Vet Parasitol. (2010) 168:231–9. 10.1016/j.vetpar.2009.11.003 19969422

[76] David ÉB Patti M Coradi ST Oliveira-Sequeira TC Ribolla PE Guimarães S. Molecular typing of *Giardia duodenalis* isolates from nonhuman primates housed in a Brazilian zoo. Rev Inst Med Trop São Paulo. (2014) 56:49–54. 10.1590/S0036-46652014000100007 24553608 PMC4085826

[77] Volotão AC Júnior JC Grassini C Peralta JM Fernandes O. Genotyping of *Giardia duodenalis* from southern brown howler monkeys (*Alouatta clamitans*) from Brazil. Vet Parasitol. (2008) 158:133–7. 10.1016/j.vetpar.2008.07.003 18834669

[78] Tan KS. New insights on classification, identification, and clinical relevance of *Blastocystis* spp. Clin Microbiol Rev. (2008) 21:639–65. 10.1128/CMR.00022-08 18854485 PMC2570156

[79] Tan KS Mirza H Teo JD Wu B Macary PA. Current views on the clinical relevance of *Blastocystis* spp. Curr Infect Dis Rep. (2010) 12:28–35. 10.1007/s11908-009-0073-8 21308496

[80] Li J Karim MR Li D Rahaman Sumon SMM Siddiki SHMF Rume FI. Molecular characterization of *Blastocystis* sp. in captive wildlife in Bangladesh National Zoo: Non-human primates with high prevalence and zoonotic significance. Int J Parasitol Parasites Wildl. (2019) 10:314–20. 10.1016/j.ijppaw.2019.11.003 31867211 PMC6906819

[81] Valença-Barbosa C do Bomfim TCB Teixeira BR Gentile R Neto SFDC Magalhães BSN. Molecular epidemiology of *Blastocystis* isolated from animals in the state of Rio de Janeiro, Brazil. PLoS ONE. (2019) 14:e0210740. 10.1371/journal.pone.0210740 30682075 PMC6347289

[82] Oliveira-Arbex AP David ÉB Tenório MDS Cicchi PJP Patti M Coradi ST. Diversity of *Blastocystis* subtypes in wild mammals from a zoo and two conservation units in southeastern Brazil. Infect Genet Evol. (2020) 78:104053. 10.1016/j.meegid.2019.104053 31683006

[83] Zhao GH Hu XF Liu TL Hu RS Yu ZQ Yang WB. Molecular characterization of *Blastocystis* sp. in captive wild animals in qinling mountains. Parasitol Res. (2017) 116:2327–2333. 10.1007/s00436-017-5506-y 28540508

[84] Helenbrook WD Whipps CM. Molecular characterization of *Blastocystis* in captive and free-ranging new world primates, Platyrrhini. Acta Parasitol. (2021) 66:1267–73. 10.1007/s11686-021-00397-1 33914238

[85] Alfellani MA Jacob AS Perea NO Krecek RC Taner-Mulla D Verweij JJ. Diversity and distribution of *Blastocystis* sp. subtypes in non-human primates. Parasitology. (2013) 140:966–71. 10.1017/S0031182013000255 23561720

[86] Deng L Yao J Chen S He T Chai Y Zhou Z. First identification and molecular subtyping of *Blastocystis* sp. in zoo animals in southwestern China. Parasit Vect. (2021) 14:11. 10.1186/s13071-020-04515-2 33407818 PMC7788908

[87] Mansfield KG Carville A Hebert D Chalifoux L Shvetz D Lin KC. Localization of persistent *Enterocytozoon bieneusi* infection in normal rhesus macaques (*Macaca mulatta*) to the hepatobiliary tree. J Clin Microbiol. (1998) 36:2336–8. 10.1128/JCM.36.8.2336-2338.1998 9666017 PMC105043

[88] Chalifoux LV Carville A Pauley D Thompson B Lackner AA Mansfield KG. *Enterocytozoon bieneusi* as a cause of proliferative serositis in simian immunodeficiency virus-infected immunodeficient macaques (*Macaca mulatta*). Arch Pathol Lab Med. (2000) 124:1480–4. 10.5858/2000-124-1480-EBAACO 11035580

[89] Sak B Kvác M Petrzelková K Kvetonová D Pomajbíková K Mulama M. Diversity of microsporidia (Fungi: Microsporidia) among captive great apes in European zoos and African sanctuaries: evidence for zoonotic transmission? Folia Parasitol. (2011) 58:81–6. 10.14411/fp.2011.008 21776888

[90] Karim MR Wang R Dong H Zhang L Li J Zhang S. Genetic polymorphism and zoonotic potential of *Enterocytozoon bieneusi* from nonhuman primates in China. Appl Environ Microbiol. (2014) 80:1893–8. 10.1128/AEM.03845-13 24413605 PMC3957649

[91] Karim MR Dong H Li T Yu F Li D Zhang L. Predomination and new genotypes of *Enterocytozoon bieneusi* in captive nonhuman primates in zoos in China: high genetic diversity and zoonotic significance. PLoS ONE. (2015) 10:e0117991. 10.1371/journal.pone.0117991 25705879 PMC4338232

[92] Yu F Wu Y Li T Cao J Wang J Hu S. High prevalence of *Enterocytozoon bieneusi* zoonotic genotype D in captive golden snub-nosed monkey (*Rhinopithecus roxellanae*) in zoos in China. BMC Vet Res. (2017) 13:158. 10.1186/s12917-017-1084-6 28583130 PMC5460354

[93] Zhao W Zhou H Jin H Sun L Li P Liu M. Genotyping of *Enterocytozoon bieneusi* among captive long-tailed macaques (*Macaca fascicularis*) in Hainan Province: high genetic diversity and zoonotic potential. Acta Trop. (2020) 201:105211. 10.1016/j.actatropica.2019.105211 31600522

[94] Karim MR Rume FI Rahman ANMA Zhang Z Li J Zhang L. Evidence for zoonotic potential of *Enterocytozoon bieneusi* in its first molecular characterization in captive mammals at Bangladesh national zoo. J Eukaryot Microbiol. (2020) 67:427–35. 10.1111/jeu.12792 32115792

[95] Lee RV Prowten AW Anthone S Satchidanand SK Fisher JE Anthone R. Typhlitis due to *Balantidium coli* in captive lowland gorillas. Rev Infect Dis. (1990) 12:1052–59. 10.1093/clinids/12.6.1052 2267484

[96] Hinde K. Milk composition varies in relation to the presence and abundance of *Balantidium coli* in the mother in captive rhesus macaques (*Macaca mulatta*). Am J Primatol. (2007) 69:625–34. 10.1002/ajp.20373 17245767

[97] Pomajbíková K PetrŽelková KJ Profousová I Petrášová J Modrý D. Discrepancies in the occurrence of *Balantidium coli* between wild and captive African great apes. J Parasitol. (2010) 96:1139–44. 10.1645/GE-2433.1 21158624

[98] Schovancová K Pomajbíková K Procházka P Modrý D Bolechová P PetrŽelková KJ. Preliminary insights into the impact of dietary starch on the ciliate, *Neobalantidium coli*, in captive chimpanzees. PLoS ONE. (2013) 8:e81374. 10.1371/journal.pone.0081374 24282589 PMC3839902

[99] Muehlenbein MP. Parasitological analyses of the male chimpanzees (*Pan troglodytes schweinfurthii*) at Ngogo, Kibale national park, Uganda. Am J Primatol. (2005) 65:167–79. 10.1002/ajp.20106 15706587

[100] Boundenga L Ngoubangoye B Moukodoum N Dibakou SE Moussadji C Hugot JP. Diversity of parasites in two captive chimpanzee populations in southern Gabon. Infect Genet Evol. (2021) 91:104807. 10.1016/j.meegid.2021.104807 33737228

[101] Pomajbíková K Petrzelková KJ Profousová I Petrásová J Kisidayová S Varádyová Z. A survey of entodiniomorphid ciliates in chimpanzees and bonobos. Am J Phys Anthropol. (2010) 142:42–8. 10.1002/ajpa.21191 19845028

[102] Profousová I Petrzelková KJ Pomajbíková K Modrý D. Survival and morphologic changes of entodiniomorphid ciliate *Troglodytella abrassarti* in chimpanzee feces. J Zoo Wildl Med. (2011) 42:69–74. 10.1638/2010-0100.1 22946373

[103] Paulos S Köster PC de Lucio A Hernández de Mingo M Cardona GA Fernández Crespo JC. Occurrence and subtype distribution of *Blastocys*tis sp. in humans, dogs and cats sharing household in northern Spain and assessment of zoonotic transmission risk. Zoonoses Public Health. (2018) 65:993–1002. 10.1111/zph.12522 30198123

[104] Muadica AS Köster PC Dashti A Bailo B Hernández-de-Mingo M Reh L. Molecular diversity of *Giardia duodenalis, Cryptosporidium* spp. and Blastocystis sp. in asymptomatic school children in Leganés, Madrid (Spain). Microorganisms. (2020) 8:466. 10.3390/microorganisms8040466 32218318 PMC7232429

