# Supplementary material for: Intestinal Protists in Captive Non-human Primates and Their Handlers in Six European Zoological Gardens. Molecular Evidence of Zoonotic Transmission
Source: Front Vet Sci. 2022 Jan 4;8:819887. doi: 10.3389/fvets.2021.819887 (PMC8763706; doi:10.3389/fvets.2021.819887)
Supplement: Supplementary file 2 [file Table_2.DOC]

**Table S2**. English version of the standardised epidemiological questionnaire used in this study.

| **EPIDEMIOLOGICAL QUESTIONNAORE FOR STAFF** |
| --- |

The nature of this epidemiological questionnaire is strictly voluntary. Data provided here will be kept confidential and used for research purposes only.

Please answer the questions as fairly as possible. Return the document together with the stool sample and the Participant Informed Consent making sure all the requested data have been filled including the name of the participant.

| Sample collection date: / / (dd/mm/yy) |
| --- |

| **DATA OF THE PARTICIPATING** |
| --- |
| Full name: |
| Gender: |
| Age: |
| Work institution: |
| Have you experienced diarrhoea in the last 7 days?  yes  no |

| **DATA ON CONTACT WITH ANIMALS AT WORK** |
| --- |
| Contact with faecal material from primates?  yes  no  Contact with faecal material from other (non-primate) animals?  yes Specify:  no  Has any of these animals presented with diarrhoea in the last 3 months?  yes  no  do not know/remember  Do you handle food intended for primates?  yes  no |

| **DATA ON CONTACT WITH PEOPLE** |
| --- |
| Contact with young (≤5 years-old) children attending kindergartens?  yes  no  ¿Has any family member presented with diarrhoea in the las month?  yes  no |

| **ENVIRONMENTAL FACTORS** |
| --- |
| Have you, or any family member, travelled abroad in the last 6 months?  yes  no  If yes, please specify visited countries: |
| Contact with dogs at home?  yes  no  Contact with cats at home?  yes  no  Has any of these animals presented with diarrhoea in the last 3 months?  yes  no  do not know/remember |
| Source of drinking water  Tap water  Bottled water  Wells  Nave you drink water from sources other than above mentioned?  yes Specify:  no |
| Did you practice any aquatic sport?  yes Specify  no |

| **PERSONAL HYGIENE PRACTICES** |
| --- |
| Hand washing before eating:  always  often  rarely |
| Washing of raw produce (salads, fruits)  always  often  rarely |
