# Supplementary material for: Intestinal Protists in Captive Non-human Primates and Their Handlers in Six European Zoological Gardens. Molecular Evidence of Zoonotic Transmission
Source: Front Vet Sci. 2022 Jan 4;8:819887. doi: 10.3389/fvets.2021.819887 (PMC8763706; doi:10.3389/fvets.2021.819887)
Supplement: Supplementary file 3 [file Table_3.docx]

**Table S3.** Oligonucleotides used for the molecular identification and/or characterization of the enteric protist species investigated in the present study.

| **Target organism** | **Locus** | **Oligonucleotide** | **Sequence (5´–3´)** | **Reference** |
| --- | --- | --- | --- | --- |
| *Cryptosporidium* spp. | *ssu* rRNA | CR-P1 | CAGGGAGGTAGTGACAAGAA | (30) |
|  |  | CR-P2 | TCAGCCTTGCGACCATACTC |  |
|  |  | CR-P3 | ATTGGAGGGCAAGTCTGGTG |  |
|  |  | CPB-DIAGR | TAAGGTGCTGAAGGAGTAAGG |  |
| *Entamoeba histolytica* | *ssu* rRNA | Probe | FAM–TCATTGAATGAATTGGCCATTT–MGB | (32) |
| *Entamoeba dispar* |  | Probe | VIC–TTACTTACATAAATTGGCCACTTTG–MGB |  |
| *Entamoeba histolytica*/*dispar* |  | Ehd-239F | ATTGTCGTGGCATCCTAACTCA | (31) |
|  |  | Ehd-88R | GCGGACGGCTCATTATAACA |  |
| *Giardia duodenalis* | *ssu* rRNA | Probe | FAM–CCCGCGGCGGTCCCTGCTAG–BHQ1 | (33) |
|  |  | Gd-80F | GACGGCTCAGGACAACGGTT |  |
|  |  | Gd-127R | TTGCCAGCGGTGTCCG |  |
|  | *gdh* | GDHeF | TCAACGTYAAYCGYGGYTTCCGT | (34) |
|  |  | GDHiF | CAGTACACCTCYGCTCTCGG |  |
|  |  | GDHiR | GTTRTCCTTGCACATCTCC |  |
|  | *bg* | G7_F | AAGCCCGACGACCTCACCCGCAGTGC | (35) |
|  |  | G759_R | GAGGCCGCCCTGGATCTTCGAGACGAC |  |
|  |  | G99_F | GAACGAACGAGATCGAGGTCCG |  |
|  |  | G609_R | CTCGACGAGCTTCGTGTT |  |
|  | *tpi* | AL3543 | AAATIATGCCTGCTCGTCG | (36) |
|  |  | AL3546 | CAAACCTTITCCGCAAACC |  |
|  |  | AL3544 | CCCTTCATCGGIGGTAACTT |  |
|  |  | AL3545 | GTGGCCACCACICCCGTGCC |  |
| *Blastocystis* spp. | *ssu* rRNA | BhRDr | GAGCTTTTTAACTGCAACAACG | (37) |
|  |  | RD5 | ATCTGGTTGATCCTGCCAGT |  |
| *Enterocytozoon bieneusi* | ITS | EBITS3 | GGTCATAGGGATGAAGAG | (38) |
|  |  | EBITS4 | TTCGAGTTCTTTCGCGCTC |  |
|  |  | EBITS1 | GCTCTGAATATCTATGGCT |  |
|  |  | EBITS2.4 | ATCGCCGACGGATCCAAGTG |  |
| *Balantioides coli* | ITS | B5D | GCTCCTACCGATACCGGGT | (39) |
|  |  | B5RC | GCGGGTCATCTTACTTGATTTC |  |
| *Troglodytella* spp. | ITS | SSU_end) | AAGGTWTCCGTAGGTGAACCTG | (40) |
|  |  | LSU_start | TAKTRAYATGCTTAAGTYCAGCG |  |

*bg*: β-giardin (bg); *gdh*: Glutamate dehydrogenase; ITS: Internal transcribed spacer; *ssu* rRNA: Small subunit ribosomal RNA; *tpi*: Triose phosphate isomerase.
