# Supplementary material for: Intestinal Protists in Captive Non-human Primates and Their Handlers in Six European Zoological Gardens. Molecular Evidence of Zoonotic Transmission
Source: Front Vet Sci. 2022 Jan 4;8:819887. doi: 10.3389/fvets.2021.819887 (PMC8763706; doi:10.3389/fvets.2021.819887)
Supplement: Supplementary file 4 [file Table_4.DOCX]

**Table S4.** Single and multiple enteric protist infections/colonisations detected in faecal samples from non-human primates in the present study.

| **Protist species combination** | **Faecal samples (*n*)** | **Relative frequency (%)** |
| --- | --- | --- |
| *Blastocystis* sp. only | 56 | 30.1 |
| *G. duodenalis* only | 58 | 31.2 |
| *E. dispar* only | 17 | 9.1 |
| *B. coli* only | 2 | 1.1 |
| *T. abrassarti* only | 6 | 3.2 |
| *E. bieneusi* only | 3 | 1.6 |
| *Cryptosporidium* spp. only | 1 | 0.5 |
| *Blastocystis* sp. + *G. duodenalis* | 15 | 8.1 |
| *Blastocystis* sp. +*E. dispar* | 13 | 7.0 |
| *Blastocystis* sp. + *B. coli* | 3 | 1.6 |
| *E. dispar* + *G. duodenalis* | 3 | 1.6 |
| *B. coli* + *Cryptosporidium* spp. | 1 | 0.5 |
| *B. coli* + *G. duodenalis* | 1 | 0.5 |
| *Blastocystis* sp. + *Cryptosporidium* spp. | 1 | 0.5 |
| *Blastocystis* sp. + *E. bieneusi* | 1 | 0.5 |
| *Cryptosporidium* spp. + *G. duodenalis* | 1 | 0.5 |
| *G. duodenalis* + *T. abrassarti* | 1 | 0.5 |
| *Blastocystis* sp. + *E. dispar* + *G. duodenalis* | 3 | 1.6 |
| Total | 186 | 100.0 |
