# Supplementary material for: Intestinal Protists in Captive Non-human Primates and Their Handlers in Six European Zoological Gardens. Molecular Evidence of Zoonotic Transmission
Source: Front Vet Sci. 2022 Jan 4;8:819887. doi: 10.3389/fvets.2021.819887 (PMC8763706; doi:10.3389/fvets.2021.819887)
Supplement: Supplementary file 5 [file Table_5.DOCX]

**Table S5.** Single and multiple enteric protist infections/colonisations detected in faecal samples from zookeepers in the present study.

| **Protist species combination** | **Faecal samples (*n*)** | **Relative frequency (%)** |
| --- | --- | --- |
| *Blastocystis* sp. only | 17 | 80.9 |
| *Cryptosporidium* spp. only | 1 | 4.8 |
| *E. dispar* only | 1 | 4.8 |
| *G. duodenalis* only | 1 | 4.8 |
| *Blastocystis* sp. + *Cryptosporidium* spp. | 1 | 4.8 |
| Total | 21 | 100 |
