# Supplementary material for: Intestinal Protists in Captive Non-human Primates and Their Handlers in Six European Zoological Gardens. Molecular Evidence of Zoonotic Transmission
Source: Front Vet Sci. 2022 Jan 4;8:819887. doi: 10.3389/fvets.2021.819887 (PMC8763706; doi:10.3389/fvets.2021.819887)
Supplement: Supplementary file 6 [file Table_6.docx]

**Table S6.** Diversity, frequency, and molecular features of *Cryptosporidium* spp. and *Giardia duodenalis* isolates identified in captive non-human primates in the present study. Institution of origin and GenBank accession numbers are provided.

| **Species** | **Species/ genotype** | **Sub-genotype** | **No. isolates** | **Institution** | **Locus** | **Reference sequence** | **Stretch** | **Single nucleotide polymorphisms** | **GenBank ID** |
| --- | --- | --- | --- | --- | --- | --- | --- | --- | --- |
| *Cryptosporidium* | *C. hominis* | Unknown^1^ | 2 | MZA, SZ | *ssu* rRNA | AF108865 | 577‒910 | None | OK285279 |
|  | *C. parvum* | Unknown^1^ | 2 | SZ | *ssu* rRNA | AF112571 | 532‒1,039 | A646G, T649G, 686-689 del TAAT, A691T | OK285280 |
| *Giardia duodenalis* | A | AII | 2 | BZ | *gdh* | L40510 | 64‒491 | None | OK318919 |
|  | B | BIV | 2 | Faunia | *gdh* | L40508 | 76‒491 | None | OK318920 |
|  | B | BIV | 1 | MZA | *gdh* | L40508 | 76‒445 | C176A^2^, T183C, T387C | OK318921 |
|  | B | BIV | 1 | MZA | *gdh* | L40508 | 76‒491 | T183Y, C311Y^3^, T387C, C468Y | OK318922 |
|  | B | BIV | 2 | Faunia | *gdh* | L40508 | 76‒496 | T183C, T387C, C396T, C432T | OK318923 |
|  | B | BIV | 1 | SZ | *gdh* | L40508 | 76‒450 | T387C | OK318924 |
|  | B | BIV | 2 | BZ | *gdh* | L40508 | 76‒435 | T387C, C432T | OK318925 |
|  | B | BIV | 1 | SZ | *gdh* | L40508 | 76‒496 | T387C, C468T | OK318926 |
|  | B | BIV | 1 | LVS | *gdh* | L40508 | 99‒491 | T387C, C468T | OL456212 |
|  | B | BIV | 1 | Faunia | *gdh* | L40508 | 76‒491 | T387Y, C468Y | OK318927 |
|  | A | AI | 1 | MZA | *bg* | AY655702 | 30‒524 | A53R^4^ | OK318928 |
|  | A | AII | 2 | BZ | *bg* | AY072723 | 103‒604 | None | OK318929 |
|  | B | B | 1 | Faunia | *bg* | AY072727 | 97‒592 | C120Y, C165Y, T306Y, C309T, C393Y, A412R^5^, C507Y | OK318930 |
|  | B | B | 1 | MZA | *bg* | AY072727 | 93‒582 | C120T, C165T, C309T | OK318931 |
|  | B | B | 1 | SZ | *bg* | AY072727 | 103‒604 | C120T, C165T, C309T, T491W^6^ | OK318932 |
|  | B | B | 1 | BZ | *bg* | AY072727 | 98‒593 | C165T, C309T, A387R, C450T | OK318933 |
|  | B | B | 1 | Faunia | *bg* | AY072727 | 133‒561 | C165T, C309T, C393T | OK318934 |
|  |  |  |  |  |  |  |  |  |  |
|  | A | AII | 1 | BZ | *tpi* | U57897 | 294‒805 | C337T^7^ | OK318935 |
|  | B | BIV | 1 | Faunia | *tpi* | AF069560 | 1‒479 | T57C^8^, T214Y^9^, A368G, A395G | OK318936 |
|  | B | BIV | 1 | BZ | *tpi* | AF069560 | 1‒448 | G161A, A176G, C347T, A368G, A395G, T404C | OK318937 |
|  | B | BIV | 1 | BZ | *tpi* | AF069560 | 52‒479 | T208C^10^, C383A^11^ | OK318938 |

*bg*: β-giardin; BZ: Barcelona Zoo; *gdh*: glutamate dehydrogenase; LVS: La Vallée de Singes; MZA: Madrid Zoo Aquarium; *ssu* rRNA: small subunit ribosomal RNA; SZ: Santillana Zoo; *tpi*: triose phosphate isomerase.

^1^ No amplification at the *gp60* locus.

^2^ pT59N

^3^ If T, pP104L

^4^ If G, p.D18G

^5^ If G, p.S138G

^6^ If A, p.V164D

^7^ p.A113V

^8^ p.Y19H

^9^ If C, p. M71T

^10^ p.V69A

^11^ p.N127K
