# Supplementary material for: Intestinal Protists in Captive Non-human Primates and Their Handlers in Six European Zoological Gardens. Molecular Evidence of Zoonotic Transmission
Source: Front Vet Sci. 2022 Jan 4;8:819887. doi: 10.3389/fvets.2021.819887 (PMC8763706; doi:10.3389/fvets.2021.819887)
Supplement: Supplementary file 7 [file Table_7.DOCX]

**Table S7.** Diversity, frequency, and molecular features of *Blastocystis* sp. isolates identified in captive non-human primates in the present study. Institution of origin and GenBank accession numbers are provided.

| **Species** | **Species/ genotype** | **Sub-**  **genotype** | **No. isolates** | **Institution** | **Locus** | **Reference sequence** | **Stretch** | **Single nucleotide polymorphisms** | **GenBank ID** |
| --- | --- | --- | --- | --- | --- | --- | --- | --- | --- |
| *Blastocystis* sp. | ST1 | Allele 1 | 5 | SZ (5) | MK357786 | *ssu* rRNA | 1‒607 | None | OK285229 |
|  | ST1 | Allele 2 | 4 | MZA (3), SZ (1) | AB107968 | *ssu* rRNA | 21‒611 | 80delG | OK285230 |
|  | ST1 | Unknown | 9 | BZ (5), SZ (4) | ‒ | *ssu* rRNA | ‒ | ‒ | ‒ |
|  | ST1 | Alleles 1+2 | 9 | SZ (5), BZ (3), MZA (1) | AB107968 | *ssu* rRNA | 18‒550 | A132R | OK285231 |
|  | ST1 | Alleles 1+2 | 1 | SZ (1) | AB107968 | *ssu* rRNA | 48‒611 | A132V | OK285232 |
|  | ST1 | Allele 3 | 1 | MZA (1) | DQ462721 | *ssu* rRNA | 62‒531 | None | OK285233 |
|  | ST1 | Allele 4 | 1 | SZ (1) | MZ396317 | *ssu* rRNA | 1‒390 | None | OK285234 |
|  | ST2 | Allele 9 | 4 | MZA (4) | MT661555 | *ssu* rRNA | 52‒554 | None | OK285235 |
|  | ST2 | Allele 11 | 1 | MZA (1) | MZ496542 | *ssu* rRNA | 1‒598 | None | OK285236 |
|  | ST2 | Allele 12 | 1 | MZA (1) | MN526752 | *ssu* rRNA | 4‒605 | G456A | OK285237 |
|  | ST2 | Allele 12 | 3 | MZA (3) | MW564223 | *ssu* rRNA | 6‒594 | A173R, A255W | OK285238 |
|  | ST2 | Allele 12 | 1 | MZA (1) | MW564223 | *ssu* rRNA | 1‒594 | A173R, A255W, A395W | OK285239 |
|  | ST3 | Allele 23 | 2 | MZA (1) | MH997480 | *ssu* rRNA | 34‒603 | 262delT | OK285240 |
|  | ST3 | Alleles 26+27 | 1 | MZA (1) | HQ909890 | *ssu* rRNA | 1‒587 | T156W, C157Y | OK285241 |
|  | ST3 | Allele 30 | 3 | MZA (3) | MZ496544 | *ssu* rRNA | 6‒588 | None | OK285242 |
|  | ST3 | Allele 34 | 1 | SZ (1) | AB107965 | *ssu* rRNA | 21‒596 | G130A, A131T, A132G, A175T, T176C, A179R | OK285243 |
|  | ST3 | Allele 37 | 1 | MZA (1) | MF184982 | *ssu* rRNA | 56‒573 | None | OK285244 |
|  | ST3 | Alleles 34+ 37 | 1 | BZ (1) | MF184982 | *ssu* rRNA | 47‒575 | C235Y | OK285245 |
|  | ST4 | Allele 42 | 4 | Faunia (4) | MN836841 | *ssu* rRNA | 3‒605 | None | OK285246 |
|  | ST5 | Allele 16 | 8 | MZA (5), BZ (3) | MT661529 | *ssu* rRNA | 3‒618 | None | OK285247 |
|  | ST5 | Allele 17 | 8 | MZA (6), BZ (1), Faunia (1) | MK801415 | *ssu* rRNA | 2‒616 | None | OK285248 |
|  | ST5 | 16+ 17 | 1 | BZ (1) | MK801415 | *ssu* rRNA | 69‒594 | C170M | OK285249 |
|  | ST5 | Unknown | 1 | MZA (1) | ‒ | *ssu* rRNA | ‒ | ‒ | ‒ |
|  | ST8 | Allele 21 | 2 | Faunia (2) | AB107971 | *ssu* rRNA | 8‒616 | None | OK285250 |

BZ: Barcelona Zoo; del: deletion; MZA: Madrid Zoo Aquarium; *ssu* rRNA: small subunit ribosomal RNA; SZ: Santillana Zoo.
