# Supplementary material for: Intestinal Protists in Captive Non-human Primates and Their Handlers in Six European Zoological Gardens. Molecular Evidence of Zoonotic Transmission
Source: Front Vet Sci. 2022 Jan 4;8:819887. doi: 10.3389/fvets.2021.819887 (PMC8763706; doi:10.3389/fvets.2021.819887)
Supplement: Supplementary file 8 [file Table_8.docx]

**Table S8.** Diversity, frequency, and molecular features of *Cryptosporidium* spp. and *Blastocystis* sp. isolates identified at the small subunit ribosomal RNA locus in zookeepers in the present study. Institution of origin and GenBank accession numbers are provided.

| **Species** | **Species/ genotype** | **Sub-genotype** | **No. isolates** | **Institution** | **Reference sequence** | **Stretch** | **Single nucleotide polymorphisms** | **GenBank ID** |
| --- | --- | --- | --- | --- | --- | --- | --- | --- |
| *Cryptosporidium* | *C. hominis* | Unknown^1^ | 1 | MZA | AF108865 | 677‒998 | 697delT | OK285278 |
|  | *C. hominis* | Unknown^1^ | 1 | MZA | AF108865 | ‒ | Unknown^2^ | ‒ |
| *Blastocystis* sp. | ST1 | Alleles 1+2 | 4 | MZA, SZ | AB107968 | 8‒611 | A132R | OK285223 |
|  | ST1 | Allele 4 | 4 | MZA, SZ | MZ396327 | 1‒390 | A374M, A375M | OK285224 |
|  | ST2 | Unknown^2^ | 2 | MZA, Faunia | ‒ | ‒ | ‒ | ‒ |
|  | ST3 | Allele 34 | 1 | BZ | MZ496545 | 27‒541 | None | OK285225 |
|  | ST3 | Alleles 12+24+27 | 1 | BZ | MN338081 | 4‒598 | A171R, A260R, T261M | OK285226 |
|  | ST3 | Allele 22 | 1 | SZ | HQ909890 | 1‒587 | None | OK285227 |
|  | ST4 | Allele 42 | 5 | Faunia, MZA | MN836841 | 1‒606 | None | OK285228 |

BZ: Barcelona Zoo; MZA: Madrid Zoo Aquarium; SZ: Santillana Zoo

^1^ No amplification at the *gp60* locus.

^2^ Sequences of insufficient quality to accuratly determine the presence of single nucleotide polymorphisms or assign sub-genotypes.
